# Excellence in Antibiotic Stewardship: A mixed methods study comparing High, Medium, and Low Performing Hospitals

**DOI:** 10.1093/cid/ciad743

**Published:** 2024-06-14

**Authors:** Valerie M. Vaughn, Sarah L. Krein, Adam Hersh, Whitney R. Buckel, Andrea T. White, Jennifer Horowitz, Payal K. Patel, Tejal N Gandhi, Lindsay A. Petty, Emily S. Spivak, Steven J. Bernstein, Anurag M. Malani, Leonard B. Johnson, Robert A. Neetz, Scott A. Flanders, Patrick Galyean, Elisabeth Kimball, Kennedi Bloomquist, Tobias Zickmund, Susan L. Zickmund, Julia E. Szymczak

**Affiliations:** 1 Division of General Internal Medicine, Department of Internal Medicine, University of Utah School of Medicine, Salt Lake City, Utah, USA; 2 Division of Health System Innovation & Research, Department of Population Health Science, University of Utah School of Medicine, Salt Lake City, Utah, USA; 3 Division of Hospital Medicine, Department of Internal Medicine, Michigan Medicine, Ann Arbor, Michigan, USA; 4 Center for Clinical Management Research, VA Ann Arbor Healthcare System, Ann Arbor, Michigan, USA; 5 Division of General Medicine, Department of Internal Medicine, Michigan Medicine, Ann Arbor, Michigan, USA; 6 Division of Infectious Diseases, Department of Pediatrics, University of Utah School of Medicine, Salt Lake City, Utah, USA; 7 Intermountain Healthcare Pharmacy Services, Taylorsville, UT, USA; 8 Division of Infectious Diseases, Department of Medicine, Intermountain Health, Salt Lake City, Utah, USA; 9 Division of Infectious Diseases, Department of Medicine, University of Michigan Medical School, Ann Arbor, Michigan, USA; 10 Division of Infectious Diseases, Department of Medicine, University of Utah School of Medicine, Salt Lake City, Utah, USA; 11 Division of Infectious Diseases, Department of Internal Medicine, Trinity Health Michigan, Ann Arbor, Michigan, USA; 12 Division of Infectious Diseases, Department of Internal Medicine, Ascension St. John Hospital, Detroit, Michigan, USA; 13 Department of Pharmacy, MyMichigan Health, Midland, Michigan, USA; 14 Division of Epidemiology, Department of Internal Medicine, University of Utah School of Medicine, Salt Lake City, Utah, USA; 15 Informatics, Decision-Enhancement & Analytic Sciences Center, VA Salt Lake City Healthcare System, Salt Lake City, Utah, USA

**Keywords:** Antimicrobial stewardship, qualitative, mixed-methods, high-performance, organizational context

## Abstract

**Background:**

Despite antibiotic stewardship programs existing in most acute care hospitals, there continues to be variation in appropriate antibiotic use. While existing research examines individual prescriber behavior, contextual reasons for variation are poorly understood.

**Methods:**

We conducted an explanatory, sequential mixed methods study of a purposeful sample of 7 hospitals with varying discharge antibiotic overuse. For each hospital, we conducted surveys, document analysis, and semi-structured interviews with antibiotic stewardship and clinical stakeholders. Data were analyzed separately and mixed during the interpretation phase, where each hospital was examined as a case, with findings organized across cases using a strengths, weaknesses, opportunities, and threats framework to identify factors accounting for differences in antibiotic overuse across hospitals.

**Results:**

Surveys included 85 respondents. Interviews included 90 respondents (31 hospitalists, 33 clinical pharmacists, 14 stewardship leaders, 12 hospital leaders). On surveys, clinical pharmacists at hospitals with lower antibiotic overuse were more likely to report feeling: respected by hospitalist colleagues (p=0.001), considered valuable team members (p=0.001), comfortable recommending antibiotic changes (p=0.02). Based on mixed-methods analysis, hospitals with low antibiotic overuse had four distinguishing characteristics: a) robust knowledge of and access to antibiotic stewardship guidance, b) high quality clinical pharmacist-physician relationships, c) tools and infrastructure to support stewardship, and d) highly engaged Infectious Diseases physicians who advocated stewardship principles.

**Conclusion:**

This mixed-method study demonstrates the importance of organizational context for high performance in stewardship and suggests improving antimicrobial stewardship requires attention to knowledge, interactions, and relationships between clinical teams and infrastructure that supports stewardship and team interactions.

## INTRODUCTION

Antibiotic overuse is common and harmful.^1,2^ While almost all acute care hospitals now have antibiotic stewardship programs,^3^ there remains a wide gap in appropriate prescribing of antimicrobials.^3–6^ Critically, improving antibiotic use requires that prescribing behavior must be understood within the complex context of hospital and team-based systems.^7–9^ However, we know little about these hospital-level contextual factors^10,11^ (e.g., social, technical, economic, cultural, organizational policy) that shape the quality of antibiotic prescribing and can inform the design and implementation of stewardship interventions. ^12–17^ Our objective was to identify contextual factors that differentiate hospitals with low vs. high antibiotic overuse.

## METHODS

### Design

We conducted an explanatory, sequential mixed methods study where hospitals were purposefully selected for in-depth study based on quantitative analysis of antibiotic prescribing data.^18^ First, we sampled 7 hospitals with variation in antibiotic overuse at discharge (see sampling below). Second, for included hospitals, we gathered survey data from antibiotic stewardship stakeholders, hospital leaders, prescribers, and pharmacists to characterize knowledge, attitudes, and practices related to antibiotic prescribing and implementation of stewardship. Third, with those same respondents, we conducted semi-structured interviews to enhance our understanding of contextual factors that may contribute to hospital antibiotic use performance. Finally, to triangulate our survey and interview data we analyzed organizational documents related to prescribing and stewardship from each hospital.^19,20^ While our sampling frame focused on antibiotic overuse at discharge, discharge prescribing is related to hospital context and stewardship culture broadly. Thus, our data collection strategies and analyses focused broadly on inpatient and discharge characteristics to identify contributions to observed differences in discharge antibiotic overuse. This project was deemed exempt by the University of Utah Institutional Review Board, and informed consent was obtained from survey and interview respondents prior to participation.

### Sampling hospitals

Hospitals from the Michigan Hospital Medicine Safety (HMS) Consortium, a 69-hospital collaborative, and from the Mountain West region of the United States were sampled based on their rates of antibiotic overuse at discharge in hospitalized patients treated for community-acquired pneumonia (CAP) or urinary tract infection (UTI). Here, we defined antibiotic overuse using a well validated metric that assesses unnecessary antibiotics, excessive antibiotic duration, and avoidable fluoroquinolones prescribed at hospital discharge.^21^ We focused on hospital discharge as it accounts for multiple prescribing decisions along a hospitalized patient’s path (e.g., diagnostic accuracy, de-escalation, duration) and varies widely across hospitals.^21^ We assessed antibiotic overuse at discharge for all 37 HMS hospitals participating in HMS between 8/1/2019 and 1/31/2020 and divided them into 3 performance tertiles: high (>1 standard deviation [SD] better than mean), medium (within 1 SD of mean), and low (>1 SD worse than mean; see eFigure 1 in appendix for details). We then purposefully sampled 1 high, 1 medium, and 1 low performing HMS hospital while targeting diverse settings (e.g., academic, community, rural). To increase geographic diversity, we also purposefully sampled a total of 4 hospitals (1 high, 2 medium, and 1 low performing) from Mountain West hospitals. Mountain West hospitals were selected in collaboration with regional stewardship leaders who identified sites they anticipated were high, medium, and low performers. We verified performance category by assessing antibiotic overuse at discharge for a random sample of approximately 100 patients with CAP or UTI per site hospitalized between 8/1/2019 – 1/31/2020. One hospital anticipated to be high performing actually fell within the “medium” performance tertile (and was analyzed as such) based on antibiotic use data. The remaining 3 Mountain West hospitals fell into their anticipated performance tertile (1 high, 1 medium, 1 low).

### Recruitment

A site liaison (typically an antibiotic stewardship program [ASP] leader) at each included hospital assisted us in identifying and recruiting four groups of respondents for surveys and interviews: a) ASP leaders; b) hospital leaders (e.g., chief quality/medical officers); c) hospitalists; and d) clinical pharmacists (i.e., non-Infectious Diseases [ID] trained pharmacists who worked with hospitalists or in care transitions). Though investigators were not blinded to hospital performance, site liaisons and study participants were blinded to their site’s performance until the conclusion of their interviews.

### Surveys

Two types of electronic surveys were administered per site (see Appendix for surveys). The first survey (one per hospital) was filled out by the site liaison to characterize antibiotic stewardship at the hospital.^22^ The second survey elicited knowledge, attitudes, and practices (KAP) of our four respondent groups (e.g., ASP leaders). KAP surveys were tailored to the professional role of the respondent and included questions eliciting demographic information, perceptions of stewardship, and perceptions of interdisciplinary interactions around prescribing and stewardship, measured on a 5-point Likert scale. The knowledge portion of the survey included 5 clinical vignettes and asked the respondent to recommend an antibiotic; vignettes included: a) typical CAP, b) CAP treated unnecessarily with broad-spectrum antibiotics, c) UTI with sepsis, d) hemodynamically stable altered mental status (unclear etiology), and e) hemodynamically unstable altered mental status (unclear etiology).

### Interviews

Semi-structured interviews were conducted over Zoom with respondents from each hospital. All interviews were conducted by an investigator with training in interview methods (VMV), using a guide informed by our prior work on antibiotic overuse.^23^ Interview guides included the same core set of questions with a subset of questions tailored to respondent’s role (see Appendix). Core questions elicited information from the respondent about their role(s), interactions with other stakeholders, hospital culture, stewardship initiatives, antibiotic prescribing resources and tools, leadership priorities, and successes and challenges related to antibiotic overuse. All interviews were audio-recorded, transcribed, and de-identified for analysis. The interviewer kept a running data collection memo for each site that recorded general impressions, recurrent themes, surprising data, discrepancies between sources, and conflicting or missing data to be clarified.

### Organizational documents

To characterize each hospital’s existing ASP guidelines, policies and tools, site liaisons provided us with organizational documents (e.g., guidelines), tools (e.g., ordersets), and resources relevant to stewardship and/or hospital discharge. The documents were triangulated with survey and interview data to enhance and confirm our understanding of each hospital’s stewardship context.^19,20^

### Data analysis

Data gathered from each hospital were analyzed separately with the point of interface (i.e., mixing) between our methods occurring during the final phase of interpretation, which forms the basis of the results presented in this manuscript.^18^ Hospital and participant survey data are presented using descriptive statistics. The association between pharmacist survey responses and hospital performance rank was assessed using Spearman correlation with p-value <0.05 considered statistically significant. Organizational documents were analyzed by a team member with expertise in antibiotic prescribing for CAP and UTI (VMV) to determine concordance of recommendations with evidence-based guidelines. Interviews were analyzed using Crabtree and Miller’s (2022) editing organizing approach to qualitative data analysis.^24^ Transcripts were uploaded to ATLAS.ti (v. 9) for management and coding by three study team members (EK, KB, TZ). A codebook was created by reviewing the transcripts and identifying concepts that emerged across interviews.^24^ These concepts were defined and discussed amongst the study team for inclusion in the final codebook. Disagreements were resolved by consensus. The final codebook was applied line-by-line to the data in ATLAS.ti with coders periodically evaluating inter-coder reliability to ensure consistency.

In the interpretive phase of analysis, we examined the survey findings, interviews (with specific attention paid to the “challenges” and “successes” coded data), and organizational documents together for each hospital, as a case. We constructed a partially ordered meta-matrix to visualize variation across cases for each data source.^25^ To populate the matrix, three investigators (VMV, JKH, ATW) conducted a case-based rapid strengths, weaknesses, opportunities, and threats (SWOT) analysis.^26^ Strengths were defined as characteristics which distinguished the hospital from peers. Weaknesses were defined as suboptimal characteristics that could represent a disadvantage in achieving high antibiotic prescribing performance or successful implementation of stewardship. Opportunities were defined as environmental conditions that could be leveraged, but were not yet, to improve antibiotic prescribing. Threats were defined as aspects of a hospital’s context that could potentially hamper progress. The SWOT approach facilitated efficient synthesis of complex information from diverse perspectives and triangulation of data from multiple sources. Through the application of SWOT, we identified strengths in low performing hospitals and weaknesses in high performing hospitals while reducing anchoring by performance.

The matrix helped identify areas of contextual variation by hospital performance status. Through multiple rounds of discussion and verification against the data we identified distinct contextual factors that varied across hospitals. Two investigators (VMV and JES) created a contrast table (Table 3) to classify the strength of each hospital on the context domain as low, medium or high.^25^ To establish trustworthiness in this process, we analyzed data in a team, examined responses both within and across sites to consider disconfirming evidence, and resolved disagreements by consensus.^27^

## RESULTS

Six of the initial seven hospitals invited to participate agreed; one medium performing HMS hospital declined and was replaced by another medium performing HMS hospital. Across the 7 included hospitals, 2 were high performing, 3 were medium performing, and 2 were low performing. In all, we interviewed 90 participants (31 hospitalists, 33 pharmacists, 14 ASP leaders, 12 hospital leaders) between April 19, 2021, and March 23, 2022 (eTables 1 and 2). Response rates were high: 90.9% (90/99) for interviews; 100% (7/7) for hospital surveys; and 95.6% (86/90) for individual surveys. Hospital characteristics are displayed in Table 1 and eTable 3. Our mixed methods analysis revealed four contextual factors that differentiated hospitals with high vs. low antibiotic overuse:

### 1) Knowledge and Comfort with Antibiotic Stewardship

We found respondent knowledge and comfort making antibiotic stewardship recommendations, particularly among clinical pharmacists, increased as performance improved.

In the knowledge vignettes, antibiotic duration and fluoroquinolone use for de-escalation decreased as performance improved (Table 2, eTable 4). Across sites, respondents chose the correct duration and antibiotic for our “easy” case vignettes (e.g., CAP); however, the harder cases showed more variation by performance. For example, the case of CAP inappropriately treated as healthcare-associated pneumonia (HCAP) provided more variable responses, with lower-performing hospitals more likely to choose excessive antibiotic duration and broader than indicated therapy when de-escalating. One explanation found via document review was that lower performing hospitals were less likely to have locally adapted guidelines or, when they had guidelines, those guidelines were either less likely to include oral antibiotic recommendations or recommended longer (e.g., 7 day) durations. For example, one medium and one low performing hospital still had guidelines referring to “HCAP” despite removal of this term in the 2019 pneumonia guidelines.^28^ Notably, the highest performing hospital had some respondents choose a 3-day duration for both CAP cases, likely due to a recent initiative at that hospital to reduce antibiotic duration to 3-days after a new clinical trial demonstrated the safety of 3-day durations for CAP.^29^ At lower performing hospitals, we also observed inconsistent answers by ASP leaders (see Table 2, eTable 4).

During interviews, we discovered that while clinical pharmacists at lower performing hospitals had similar time in practice to their counterparts at higher performing facilities, they had less post-graduate and stewardship-specific training. They also reported less access to ID physicians or institution-specific guidelines to answer questions; instead, clinical pharmacists at lower performing hospitals were more likely to report they referred to references outside their system (e.g., regional antibiograms).

**Figure 1.**
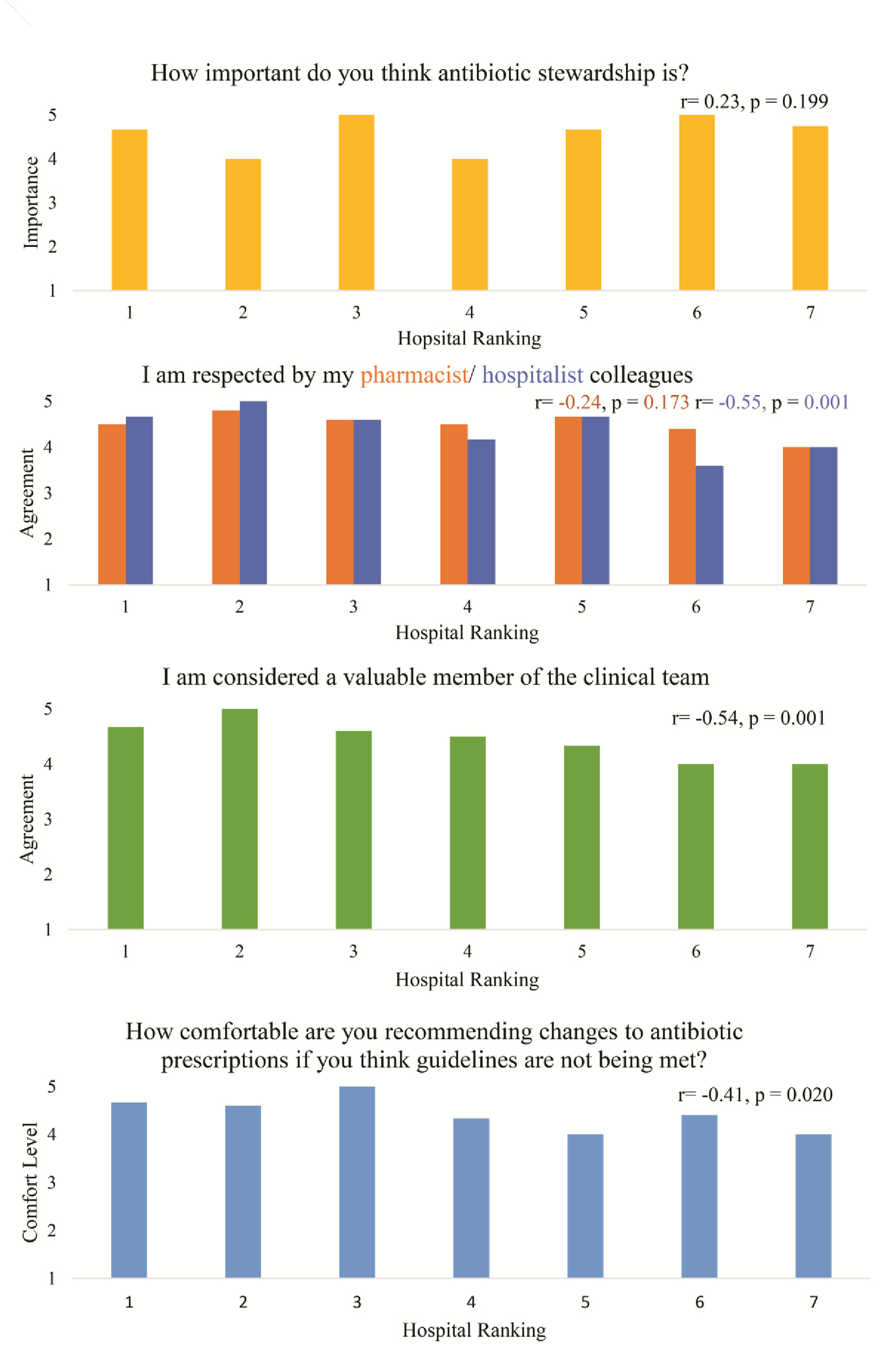
Association of Pharmacist Survey Responses with Hospital Ranking in Discharge Antibiotic Use, n=34 pharmacist respondents On surveys, pharmacists at hospitals with lower antibiotic overuse at discharge were significantly more likely to strongly agree that they felt respected by their hospitalist colleagues, strongly agree they were considered a valuable member of the clinical team, and report they were very comfortable speaking up to recommend antibiotic changes. Data were obtained via pharmacists’ surveys with all answers assessed using a 5-point Likert scale (1=very unimportant/strongly disagree/very uncomfortable, 5=very important/strongly agree/very comfortable). Mean survey responses are shown by hospital ranking defined by the amount of antibiotic overuse (where 1=lowest antibiotic overuse, 7=most antibiotic overuse). The association between pharmacist survey responses and hospital performance rank was assessed using Spearman correlation with p-value <0.05 considered statistically significant. See eTable 5 for more details.

In surveys (see Figure 1, eTable 5), we found pharmacists’ reported comfort making antibiotic recommendations decreased as hospital performance worsened (p=0.02). During interviews, clinical pharmacists at high performing hospitals indicated they were comfortable making antibiotic recommendations and tended to describe stewardship as a key component of their job.

Taken together, these findings indicate that knowledge about optimal antibiotic use matters for performance. The level of knowledge key stakeholders had in our study was driven by training; availability, accuracy, and specificity of local guidelines; and access to ID expertise.

### 2) Inter-professional Dynamics/Group Cohesiveness

Examination of answers by different stakeholders during interviews and clinical pharmacist survey responses suggested that high performing hospitals had more cohesive, team-based groups. In interviews, clinical pharmacists at high performing hospitals noted less “pushback” from clinicians about their antibiotic recommendations than did medium or low performers. When there were disagreements, hospitalists at high performing hospitals tended to be described as (or describe themselves as) more open minded. Clinical pharmacists at high performing hospitals were more likely to report trying to resolve disagreements about antibiotic stewardship recommendations by bringing evidence-based literature to the discussion. As one clinical pharmacist at a high performing hospital stated:

“It kind of varies with the personality of the provider. Sometimes, I will bring data to them to explain my recommendation, where I am coming from. Other times, if it’s not a big deal, I don’t push the recommendation very much. If I think it’s important I will definitely re-address that recommendation with either more information or find a different approach to bring it up, maybe find more data. But, oftentimes, we are able to kind of discuss the topic together.”

When surveyed, clinical pharmacists at lower performing hospitals reported feeling less respected by hospitalists and less valued as members of the clinical team (p=0.001 and p=0.001, respectively; Figure 1, eTable 5). This was confirmed during interviews when clinical pharmacists at lower performing hospitals described “picking their battles” when considering whether to engage with hospitalists about antibiotic decisions. They described hospital cultures with absolute respect for “physician autonomy” that decreased their willingness to speak up, as one clinical pharmacist at a low performing hospital explained:

“Truthfully, I’m not going to lie. There are times where I see stuff that’s wrong and I’m just like, forget it… if it’s not going to hurt anybody…if it was a couple of [antibiotic] days.”

Structural aspects of hospital context may have weakened cohesiveness. High performing hospitals were more likely to have face-to-face interactions between hospitalists and clinical pharmacists, usually during rounds or during scheduled in-person periods to “run the list.” In-person interactions were viewed as the optimal way to engage about antibiotic prescribing across performance groups. Respondents felt that it allowed for bidirectional learning, back-and-forth communication, and development of relationships. It also enabled clinical pharmacists to identify discharges before they happened to improve discharge antibiotic prescribing. Two lower performing hospitals required clinical pharmacists to go through an intermediary (e.g., nurse coordinator) to make recommendations to hospitalists, resulting in a “game of telephone” and limiting dialogue and feedback:

“We had a patient whose cultures drawn in the ER came back with [Extended Spectrum Beta-Lactamase], the patient was only on Ceftriaxone and so I called the team first thing in the morning, asked to switch to a Carbapenem and the nurse coordinator either didn’t understand – anyway, I don’t know, the Carbapenem didn’t get ordered and so I had to check back with the physician a couple of hours later, so that did delay starting the right antibiotic…”

Given the emphasis all groups placed on “discussion” and “conversation” as a mechanism for conflict resolution and learning, the lack of opportunities for face-to-face engagement was seen as a threat to relationship-building and knowledge exchange.

Another noted structural barrier to group cohesiveness was the presence of private physicians—either hospitalists or ID—who had external reporting structures. These private clinician groups, found in lower performing hospitals, were described as harder to engage in quality or stewardship initiatives. For example, a pharmacist at a medium performing hospital reported:

“It’s just recently that we’re able to find out which physicians are not following the guidelines… and we’re finding that it’s our [private] hospitalists that are more of the culprit. And those are the hardest group that we have not been able to get into a room to have conversations with…they don’t have regular meetings.”

High performing hospitals had also implemented structural facilitators of clinician engagement. One high performing hospital, for example, included antibiotic stewardship metrics and meeting participation in their bonus structure for hospitalists.

Taken together, these data suggest that group cohesiveness—particularly between clinical pharmacists and hospitalists—promotes lower antibiotic overuse and structures within hospital context can either promote or hinder these relationships.

### 3) Tools and Infrastructure

Infrastructure to support ASP and the strength of stewardship interventions differed across performance. In interviews, clinical pharmacists from high performing hospitals spoke of robust ASPs and resources which helped them feel comfortable and supported in making antibiotic recommendations, a finding confirmed in hospital surveys which demonstrated more stewardship interventions and more support for ID physician and ID pharmacist time to engage in antibiotic stewardship at high performing hospitals (Table 1). Beyond just having more interventions, we also found guidelines were more robust and better integrated across the institution in high performing hospitals. An example of thorough integration was observed at one high performing hospital, where antibiotic stewardship guidelines were used as a tool by pharmacists in their daily work and informed the design of decision-support tools used by the hospitalists. This integration was important as no hospitalists, regardless of hospital performance, reported using institutional guidelines; rather, they used built in institutional tools (e.g., decision support) when available, referred to UpToDate,^30^ or trusted their existing knowledge. Instead, institutional guidelines were helpful for clinical pharmacists to point to as standard of care if hospitalists disagreed with their recommendations.

Another key element for tools and infrastructure was engagement of hospitalists in their development. Though all hospitals reported having decision-support tools for CAP, we found hospitalists were only aware of the decision-support tools at high performing hospitals where they had been engaged in tool design and implementation. Our document analysis revealed that clinical decision-support tools at high performing hospitals were more detailed (e.g., had oral de-escalation options) and accurate (e.g., avoiding multidrug resistant organism coverage for most CAP) than at lower performers. Thus, higher performing hospitals were not only more likely to have stewardship tools, but they had high quality tools and engaged hospitalists in their design. Notably, one of our smaller, lower performing hospitals was beginning a program to address ASP needs within their size constraints through tele-ID/ASP. The feedback and excitement from local pharmacists and hospitalists about these soon-to-be resources was universally positive.

In addition to stewardship infrastructure, high performing hospitals reported more non-stewardship specific pharmacy infrastructure such as a “meds to bed” program (i.e., delivery of discharge medications to bedside) and/or transition of care pharmacists (Table 1). One leader at a high performing hospital described spending years building the business case for these programs:

“How we got funding to do it was starting kind of a formal meds to beds program so the revenue from capturing those prescriptions helped fund the pharmacists’ time to…do the discharge medication reconciliation. Now, we do that for all patients, whether they are filling their prescriptions with us or not, but that was how the finances worked out enough so that we could provide that clinical service.”

In contrast, two medium and one low performing hospital had recently closed their on-site outpatient retail pharmacies in favor of expansion in other areas, creating a gap in ability to fill or error-check medications prior to discharge.

### 4) ID Physician Support and Engagement

While national guidelines recommend ASPs be co-led by an ID physician and ID pharmacist,^3^ this was not feasible for all hospitals as not all hospitals had access to ID pharmacists. Notably, we found variation in quantitative and qualitative ID physician leadership and engagement across performance. The lowest-performing hospital in our sample had a private ID physician with minimal stewardship FTE that, according to interview respondents, was too clinically busy. Despite their assigned role as an ASP leader, it was reported they did not participate in stewardship meetings and, when consulted, often made clinical recommendations contrary to stewardship principles. In contrast, the 2 high performing hospitals had strong ID physician leadership (with more reported FTE support). While the medium performing hospitals had strong ID leadership, engagement of remaining ID physicians was mixed. One medium performing hospital had very high engagement: all ID physicians were required to be on the stewardship team and ID consults were used as stewardship interventions. In contrast, another medium performing hospital had multiple ID groups including a private group viewed as contentious and disagreeable, making stewardship when that group was on difficult:

“You know…one of them when I call, gets very angry and seems quite put out that I am talking to [them] in the first place. There’s one I don’t necessarily love their recommendations… just feeling like they don’t do quite what I would usually expect an ID doc to recommend… And then there’s one who you feel like you are [insignificant] when you call them…So, if I need to call those people, I do but if it’s like… [sighs] yeah, I can figure it out on my own and I just needed a pat on the back, I won’t necessarily reach out.”

At some hospitals, ID physicians expressed a desire to be more involved but reported having less institutional power. For example, an ID physician at one medium performing hospital wanted to change pneumonia guidelines to reflect updated evidence about shorter durations of therapy but could not because pulmonologists were perceived to “own” the pneumonia guideline and disagreed with the change.

Clinical pharmacists described relying on ID physicians to provide back-up for difficult scenarios. This was particularly true for hospitals where physician autonomy was described as a predominant value characterizing hospital culture and in one hospital where substantial gender imbalances existed between the pharmacists (predominantly female) and hospitalists (predominantly male). In these hospitals, pharmacists noted that an “MD to MD” discussion was often necessary to change clinical practice:

“I wish there wasn’t resistance among some providers, but it just seems like there is some resistance and so…we have escalated, actually, a couple of things recently that we thought were more safety [than stewardship] issues…So, getting a physician involved that is usually our next step…because, unfortunately, sometimes [the physician is] more respected than us.”

Taken together, these findings suggest the importance of ID physician support, engagement, and availability to promote high prescribing performance. Though FTE and institutional support for ID physicians is necessary, it is insufficient. For hospitals with multiple ID physicians or physician groups it is critical that all ID physicians (not just the ASP leader) are supportive of antibiotic stewardship principles and are respected and approachable leaders. In contrast, we found variation in access to ID pharmacists but not engagement with or support of antibiotic stewardship principles by ID pharmacists across performance categories.

### Summary of Findings

The results of our mixed-methods integration process displaying our 4 main hospital context findings are shown in Table 3 (details in eTable 6).

## DISCUSSION

Based on surveys, document analysis, and interviews with 90 stakeholders across 7 hospitals, we found four contextual factors that varied between hospitals with high vs. low antibiotic overuse: robust knowledge of and access to antibiotic stewardship guidance, high quality clinical pharmacist-physician relationships, tools and infrastructure to support stewardship, and highly engaged Infectious Diseases physicians who advocated for stewardship principles.

Our study confirms prior findings that demonstrate the importance of organizational context on hospital performance generally.^11,31–33^ While most efforts to improve antibiotic prescribing in hospitals focus on the behavior of individual clinicians with little emphasis on the social context surrounding this behavior,^8,34–36^ our study demonstrates how targeting individual behavior or knowledge is likely insufficient to improve antibiotic use. Relationships and the roles that stewardship stakeholders play also matter. ^12–14^Efforts to improve how decisions are made about antibiotics require attention to the knowledge, interactions between stakeholders, and systems in place to support stakeholders. Prior studies have found “handshake stewardship” with face-to-face interactions between stewards and prescribers can improve stewardship efforts.^37^ In our study, only 1 hospital in our study had direct interactions between the ASP team and clinicians; in the remainder, frontline clinical pharmacists functioned as “stewardship extenders” making antibiotic recommendations in day-to-day interactions with the clinical team. For these “stewardship extenders,” face-to-face interactions were critical for improving relationships. Furthermore, the comfort of clinical pharmacists in promoting antibiotic stewardship varied based on the context and robustness of ASP interventions, support by and availability of the ASP team, and documents to guide ASP recommendations. While not all aspects of hospital infrastructure can be changed,^38–41^ our study offers insights about ways hospitals can improve aspects of their organizational context to support more appropriate antibiotic use by supporting the teams making antibiotic decisions.

It is important to acknowledge the challenging resource constraints faced by our lowest performing hospitals. First, our low performing hospitals tended to be smaller hospitals and not the “flagship” facilities for their region. This meant they had fewer resources and less access to expertise. Prior studies have found tele-stewardship and remote ID consultation to be effective ways to help hospitals without on-site ID clinicians.^42–44^ We found local pharmacists and hospitalists were appreciative of and excited about these tele-programs. Second, one of our low and all medium performing hospitals employed private physician groups. Barriers for private physician engagement included higher patient volumes, volume-based incentives (created by the group, not the hospital), lack of consistent/well-attended meetings, and seeing patients at multiple facilities. No stakeholder reported success engaging private hospitalist or ID physicians in antibiotic stewardship, demonstrating the need for further work in these contexts. The most important lesson to come from our study is that innovative strategies are needed for supporting under-resourced hospitals as one size does not fit all when it comes to improving a complex process like antibiotic prescribing.

Our study has limitations. First, lower performing hospitals had a lower response rate, which could introduce nonresponse bias. Second, our low performers were under-resourced, limiting our ability to understand how high performance could be promoted despite resource constraints. Third, we only included 7 hospitals and our findings may not be generalizable to other contexts. Fourth, though we avoided conducting interviews during COVID19 surges, all interviews and surveys were conducted at various stages during the pandemic. While all hospitals reported the pandemic impacted ASP and face-to-face interactions, we did not explore this specifically. Fifth, the interviewer was not blinded to the hospital’s performance. We minimized the impact of any preconceptions on data collection by ensuring consistent use of the same interview guide across sites, blinding respondents, and specifically reviewing for strengths in low performers and weaknesses in high performers. Our study also has strengths including high response rates, inclusion of diverse hospitals in terms of patient populations and infrastructure, and triangulation of multiple data sources to produce a more complete understanding of hospital context.

Based on our findings, there are four major recommendations for encouraging high performance in antibiotic prescribing. First, maintain updated and accurate clinical guidelines including details for oral de-escalation with matching decision-support (or similar tools tailored to setting) for common infectious conditions. Even if guidelines are not used by hospitalists, they serve as a valuable resource for clinical pharmacists to increase/standardize their knowledge and provide an institutional reference to increase their power in conversations with frontline clinicians. Second, find ways to promote face-to-face interactions between clinical pharmacists and physicians (e.g., daily rounds). This may improve bi-directional learning, communication quality, and strengthen relationships which may promote an environment where discussions can be held openly about antibiotic use. Third, engage end-users in the design and implementation of stewardship tools, such as guidelines and ordersets, which can improve their adoption by hospitalists. Finally, provide institutional support for ASP (including dedicated effort for ID pharmacists and physicians) and ensure all ID physicians and ID physician groups are engaged in, or at least supportive of, the goal of improving antibiotic use.

In conclusion, we found organizational context was important for high performance in stewardship. A combination of knowledge of evidence-based stewardship principles, dedicated resources and infrastructure, strong social relationships between stakeholders facilitated by in-person interactions, and advocacy by Infectious Diseases physicians differentiated high from low performing hospitals. These findings suggest improving antimicrobial stewardship performance requires attention to the knowledge, interactions, and relationships between clinical teams as well as an infrastructure that supports stewardship and across-team interactions.

## Supplementary Material

Supplementary

## Figures and Tables

**Table 1.** Hospital Characteristics by Performance Category

	Hospital #1: High Performing	Hospital #2: High Performing	Hospital #3: Medium Performing	Hospital #4: Medium Performing	Hospital #5: Medium Performing	Hospital #6: Low Performing	Hospital #7: Low Performing
**Hospital Characteristics**							
General Description^a^	Academic medical center	Community medical center	Community medical center	Community medical center	Community hospital	Rural hospital	Community hospital
RUCC Score / Setting^b^	1/Large Central Metro	2/Medium Metro	1/Large Central Metro	1/Large Central Metro	2/Medium Metro	7/Rural	3/Small Metro
Bed size^c^	>600	300–600	300–600	300–600	<300	<300	300–600
Inclusion in Larger System	No	Yes, National	Yes, Regional	Yes, National	Yes, Regional	Yes, State	Yes, Regional
Stewardship on-site	Yes	Yes	Yes	Yes	Yes	No	Yes
Stewardship relationship to larger system	N/A	Flagship for stewardship in region	Flagship for stewardship in region	Flagship for stewardship in region	Tele-stewardship support	Stewardship oversight from flagship	Some stewardship support from system
**Hospitalist and ID Program Characteristics**							
Number of Hospitalist Groups	Single	Multiple (2 groups)	Multiple (2 groups)	Multiple (2–3 groups)	Multiple (2 groups)	Single	Single
Hospitalist Employer	Hospital; Some work at multiple hospitals	Hospital or nearby hospital; Some work at multiple hospitals	Hospital or private; Some work at multiple system hospitals	Hospital or private; Some with outpatient duties	Hospital or private; Some with outpatient duties	Private with reporting structure separate from hospital/system	Hospital
Hospitalist Program Size/Description	~30; see 12–16 patients daily; do not provide ICU care	~60; see 14–20 patients daily; do not provide ICU care	~35; see 16–20 patients daily	10–20; see 10–20 patients daily including ICU	~7 hospitalists; see 12–16 patients daily	~10 hospitalists; see 12–15 patients daily including ICU	28 hospitalists; see 16 patients daily including ICU
Hospitalist Incentive structure	None	Quality (group metric includes stewardship), participation in group activities; 2/6 reported productivity too	None	RVU based; half (2/4) reported having QI metrics too	None	RVU based	None
ID infrastructure	Large on-site ID consulting service	Large on-site ID consulting service	Large on-site ID consulting service; mix of hospital and private physicians	Large on-site ID consulting service	Easy access to tele-ID consults	Generally, unavailable; recently hired system ID physician for tele-ID consults	Single, very busy on-site ID physician
**Percent of Patients with Antibiotic Overuse at Discharge (time period: 8/1/2019 – 1/31/2020)**							
Main contributor to antibiotic overuse	N/A, outstanding performance	N/A, outstanding performance	Excess duration for CAP; Treating ASB & excess duration for UTI	Excess duration for CAP	Suboptimal fluoroquinolone use	Excess duration for CAP; ASB treatment	Excess duration for CAP; all types of overuse for UTI, particularly duration
Overall	8.5% (14/164)	9.9% (14/142)	29.3% (29/99)	30.6% (45/147)	38.6% (34/88)	51.3% (77/150)	68.6% (70/102)
CAP	5.6% (5/89)	14.5% (11/76)	29.4% (15/51)	30.6% (24/74)	44.0% (22/50)	58.6% (58/99)	66.0% (33/50)
UTI	12.0% (9/75)	4.6% (3/66)	29.2% (14/48)	28.8% (21/73)	31.6% (12/38)	37.3% (19/51)	71.2% (37/52)
**ASP Leadership Characteristics**							
Stewards (FTE)							
ID Pharmacist	Yes (1.25)	Yes (1.0 for 2 hospitals, majority done locally)	Yes (2.0); also covers ID consults	No; does education not direct stewardship	Yes (1.85 tele for 16 hospitals); no local	System Level (0.5 FTE for 6 hospitals); no local	Yes (1.0); recently hired
ID Physician	Yes (0.50)	Yes (0.2)	Yes (0.3)	Yes (0.20)	Minimal (0.05 for 16 hospitals), no local	System Level (0.1 FTE for 6 hospitals); no local	Minimal (0.05 FTE)
Pharmacist (not ID)	No	No	No	Yes (0.50), has stewardship training	Yes (no FTE)	No	No
Physician (not ID)	No	No	No	No	Yes (no FTE)	No	No
**Number of ROAD Home Interventions** ^d^							
Tier 1 (Critical Infrastructure)	4	5	6	4	4	3	3
Tier 2 (Inpatient Directed)	12	10	13	8	13	12	4
Tier 3 (Discharge Specific)	2	2	1	0	0	0	0
Sum	18	17	20	12	17	15	7
**Hospital Organizational Characteristics**							
Clinical pharmacists included in rounds?	Yes, on resident teams; for non-resident teams “run the list” in the afternoon	Variable, round in ICU, geriatric, and teaching teams; on other services may attend interdisciplinary rounds	Yes, on teaching teams, Otherwise no	No (sometimes pharmacy students)	No	No, except ICU teams	No
Pharmacist to hospitalist communication	Daily rounds (face-to-face teaching teams only) or afternoon “running the list” (face-to-face or phone)	Daily rounds or interdisciplinary rounds. Otherwise, throughout day via phone, messaging	Daily rounds (face-to-face teaching teams only); as needed communication on non-teaching teams either through a nurse coordinator or via phone/text	No standardized process; hospitalists will reach out if question	Through nurse coordinator (pharmacist to nurse to hospitalist)	Mostly phone/text; Face-to-face only in ICU daily rounds	Mostly phone, text; often through a midlevel/nurse coordinator; each pharmacist works with multiple hospitalists
Stewardship to hospitalist team communication style	Usually through clinical pharmacist. If escalated, note left in chart.	Rarely direct, only if escalated by clinical pharmacist	Handshake rounds with teaching teams 3x/week; none with non-teaching teams	No direct; if needed, escalates through ID consult	Rarely direct; usually through clinical pharmacist	Indirect only via local clinical pharmacist	N/A (only recently hired)
**Pharmacy Resources**							
Meds to Bed Program^e^	Yes	Yes	No	Yes	No	Yes	No
TOC pharmacist	Yes	High-risk patients only (0.8 FTE)	No	High-risk patients only	No	No	No
**Interviewees - Participation Rate (participated/invited)**							
Response Rate	100% (16/16)	100% (15/15)^f^	88% (15/17)^f^	93% (14/15)^f^	73% (8/11)^f^	100% (15/15)	70% (7/10)^f^

Unless described below, data were obtained from hospital or stakeholder surveys.

Abbreviations: RUCC, rural-urban classification code; ICU, intensive care unit; APC, advanced practice clinician; RVU, relative value unit (i.e., productivity-based incentive); QI, quality improvement; ID, infectious diseases; CAP, community-acquired pneumonia; ASB, asymptomatic bacteriuria; UTI, urinary tract infection; ASP, antimicrobial stewardship program; FTE, full-time equivalent; ROAD Home, Reducing Overuse of Antibiotics at Discharge Home; ICU, intensive care unit; TOC, transitions of care

a Hospital Information obtained from Medicare’s “Hospital General Information” 4/28/2023 (https://data.cms.gov/provider-data/dataset/xubh-q36u).

b Urban-Rural Classification Scheme for Counties. National Center for Health Statistics. Vital Health Stat 2(166). 2014. A score of ≥4 is considered rural with a score of 7–9 indicating very rural.

c Hospital bed size was obtained from the 2019 Michigan Certificate of Need Annual Survey, hospital websites, or hospital report. Numbers provided as ranges to protect confidentiality.

d Antibiotic stewardship interventions as described in the ROAD Home model where Tier 1 is Critical Infrastructure (e.g., dedicated stewardship resources, local guidelines), Tier 2 is Broad Inpatient Interventions (e.g., audit & feedback, antibiotic timeout), and Tier 3 is Discharge-Specific Strategies (e.g., discharge intervention de-emphasizing fluoroquinolones, audit & feedback of discharge antibiotics).

e A pharmacy program where discharge medications are filled and delivered to bedside prior to patient discharge.

f Not everyone interviewed from this hospital responded to surveys (see eTable 1 in appendix for details).

**Table 2.** Summary of Vignette Responses, by Hospital Performance

	High Performing, n=14	High Performing, n=12	Medium Performing, n=12	Medium Performing, n=12	Medium Performing, n=5	Low Performing, n=13	Low Performing, n=6	P-value
Total Correct Responses	86/102 (84.0%)	71/90 (78.9%)	78/95 (82.1%)	76/94 (80.9%)	28/38 (73.7%)	85/104 (81.7%)	29/45 (64.4%)	0.152^a^
Discharge Duration, mean (SD)	1.2 (1.5)	1.8 (1.5)	2.2 (1.7)	2.2 (1.9)	1.8 (2.6)	1.9 (1.5)	2.8 (3.1)	0.003^b^
Fluoroquinolone Use (%)	2/70 (2.9%)	5/60 (8.3%)	3/60 (5.0%)	3/60 (5.0%)	2/25 (8.0%)	2/65 (3.1%)	5/30 (15.7%)	0.190^a^
ASP Consistency: % of Vignettes in which ASP Leaders Had Same Answer	4/5 (80%)	3/5 (60%)	1/5 (20%)	3/5 (60%)	1/5 (20%)	1/5 (20%)	Unable to Assess (only 1 ASP)	0.274^c^

Summary responses—shown by performance—for 5 case-based vignettes on urinary tract infection or pneumonia asked to antibiotic stewards, clinical pharmacists, and hospitalists. Hospitals with more antibiotic overuse at discharge tended to have responses suggesting longer discharge durations. Antibiotic stewards at high performing hospitals often recommended the exact same discharge antibiotic and duration. For details on answers by vignette and by respondent type, please see eTable 4.

P-values for:

a Chi-square test;

b One-way ANOVA; and

c Fisher’s exact test

Abbreviations: SD, standard deviation; ASP, antibiotic stewardship program

**Table 3.** Summary of Findings by Hospital Context and Discharge Antibiotic Prescribing Performance

Hospital #	Knowledge of and Access to Antibiotic Stewardship Guidance	Inter-professional Dynamics/Group Cohesiveness	Antibiotic Stewardship Tools and Infrastructure	ID Physician Support for and Engagement with Stewardship	Antibiotic Prescribing Performance
Hospital 1	■ ■ ■	■ ■ ■	■ ■ ■	■ ■ ■	High 🠥
Hospital 2	■ ■ ■	■ ■ ■	■ ■ ■	■ ■ ■	High 🠥
Hospital 3	■ ■ ■	■ ■	■ ■ ■	■ ■	Mid 🠦
Hospital 4	■ ■	■ ■	■ ■	■ ■ ■	Mid 🠦
Hospital 5	■	■	■ ■ ■	■ ■	Mid 🠦
Hospital 6	■ ■	■	■ ■	■ ■	Low 🠧
Hospital 7	■	■	■	■	Low 🠧

■ Low

■ ■ Medium

■ ■ ■ High

## Data Availability

Due to the inability to completely de-identify qualitative data and because respondents did not consent to re-use of their data outside of this study, data are not publicly available.
