# Supplementary material for: Excellence in Antibiotic Stewardship: A mixed methods study comparing High, Medium, and Low Performing Hospitals
Source: Clin Infect Dis. Author manuscript; Available in PMC 2024 Jun 14. (PMC11153329; doi:10.1093/cid/ciad743)
Supplement: Supplementary [file NIHMS1957500-supplement-Supplementary.docx]

Appendix

[1. Quality Improvement Survey 2](#_Toc145946736)

[2. Stakeholder Surveys 6](#_Toc145946737)

[2.1 Clinical Vignettes 6](#_Toc145946738)

[2.2 Pharmacist Survey 11](#_Toc145946739)

[2.3 Hospitalist Survey 16](#_Toc145946740)

[2.4 Antibiotic Stewardship Leader Survey 21](#_Toc145946741)

[2.5 Hospital Leadership Survey 24](#_Toc145946742)

[3. Interview Guides 26](#_Toc145946743)

[3.1 Pharmacist Interview Guide 26](#_Toc145946744)

[3.2 Hospitalist Interview Guide 30](#_Toc145946745)

[3.3 Antibiotic Stewardship Leader Interview Guide 33](#_Toc145946746)

[3.4 Hospital Leadership Interview Guide 38](#_Toc145946747)

[4. Antibiotic Overuse 41](#_Toc145946748)

[4.1 eFigure 1. Antibiotic Overuse at Hospital Discharge – Community-Acquired Pneumonia and Urinary Tract Infection Combined 41](#_Toc145946749)

[eTable 1. Interview and Survey Response Rates, by Hospital 42](#_Toc145946750)

[eTable 2. Interview Responder Characteristics, by Hospital 43](#_Toc145946751)

[eTable 3. Antibiotic Stewardship Characteristics and Infrastructure, by Hospital Performance 44](#_Toc145946752)

[eTable 4. Summary of Vignette Responses, by Hospital Performance 48](#_Toc145946753)

[eTable 5. Pharmacist Survey Responses, by Hospital Performance 52](#_Toc145946754)

[eTable 6. Joint Display Describing 4 Major Themes from the Mixed-Methods Data Integration 53](#_Toc145946755)

# Quality Improvement Survey

The purpose of this study is to learn more about your hospital's approach to antibiotic prescribing. We hope this research will be useful in helping to identify ways to improve antibiotic use.

The risks of participating in this study are minimal. We will not ask you about sensitive topics, but it is possible that you may feel uncomfortable answering some questions; you may choose not to answer. There is a small risk of loss of confidentiality. All data for this project will be stored on secure computer systems. The information collected in this research will not be used for future studies.    
 
You may contact the IRB if you have questions regarding your rights as a research participant. Also, contact the IRB if you have questions, complaints or concerns which you do not feel you can discuss with the investigator. The IRB may be reached by phone at …

By completing the information below, you are giving your consent to participate in this research. Thank you for your willingness to participate.

**General Stewardship**

On January 1, 2017, the Joint Commission launched a new standard for hospitals, critical access hospitals and nursing care centers that addresses antimicrobial stewardship. Since January 1, 2017, have stewardship resources increased at your hospital (i.e., additional FTE support, etc.)?

- Yes
- No

Does your hospital have a policy that requires prescribers to document the following in the daily progress notes and/or discharge summary (Check all that Apply)?

- Dose
- Intended Duration of Use
- Indication
- None of the above

Does your facility have antibiotic use data on antibiotics prescribed at discharge?

- Yes
- No

If Yes, please describe the antibiotic use data on antibiotics prescribed at discharge.

________________________________________________________________

________________________________________________________________

At your facility, is there a review of outpatient antimicrobial therapy orders prior to discharge?

- Yes
- No

Does your hospital have a formal procedure/policy for reviewing the appropriateness of all antibiotics after the initial orders (e.g., a "timeout" 48-72 hours after starting antibiotics)?

- Yes
- No

Does your stewardship program perform audit and feedback on patients with any of the following? (Select all that apply)

- Pneumonia (CAP or HCAP)
- Asymptomatic Bacteriuria (ASB)
- Urinary Tract Infection (UTI)
- None of the above

**Stewardship – Pneumonia**

Does your hospital have a preset duration of antibiotics for patients with pneumonia?

- Yes
- No

Do you have an automated process for discharge antibiotic duration for patients with CAP?

- Yes
- No

Does your stewardship program provide education to clinicians and other staff on improving antibiotic prescribing for patients with pneumonia?

- Yes
- No

Does your computerized physician order entry (CPOE) contain order sets specific for patients with pneumonia which incorporates your facility-specific treatment recommendations?

- Yes
- No

If Yes, select which groups you provide education on pneumonia (select all that apply).

- Physicians
- ED Physicians
- Residents and fellows
- Nurses
- ED Nurses
- Advanced Practice Providers (NP or PA)
- ED Advance Practice Providers
- Patients and/or families
- None of the above

**Stewardship – UTI**

Does your computerized physician order entry (CPOE) contain decision support to discourage the attainment of urine culture in asymptomatic patients?

- Yes
- No

What interventions to reduce inappropriate use of urine cultures (e.g., diagnostic stewardship) has your hospital started?

- Removal/change in urine culture testing from preoperative order sets
- Removal/change of urine culture testing from ED order sets
- Removal/change of urine culture testing from admission order sets
- Removal of urine culture testing from other order sets
- Added reflex testing (urinalysis cutoff to urine cultures)
- Removed reflex testing (urinalysis cutoff to send urine cultures)
- Hiding urine culture results in some settings
- Requiring physician order to run urine cultures in ED
- Other two-step urine culture initiative to reduce urine cultures in ED
- Framing urine culture results in test results (e.g., adding language about asymptomatic bacteriuria)
- Rejection of some urine cultures (e.g., based on squamous cells)
- Other

Does your stewardship program provide education to clinicians and other staff on improving antibiotic prescribing for patients with UTI and Asymptomatic Bacteriuria (ASB)?

- Yes
- No

If yes, select which groups you provide education on UTI and Asymptomatic Bacteriuria (ASB) (select all that apply).

- Physicians
- ED Physicians
- Residents and fellows
- Nurses
- ED Nurses
- Advanced Practice Providers (NP or PA)
- ED Advance Practice Providers
- Patients and/or families
- None of the above

**Stewardship – Fluoroquinolones**

Which of the following Fluoroquinolones are able to be prescribed without prior approval or prospective audit and feedback (i.e., not restricted)

- Ciprofloxacin (Cipro, Ciproxin, Ciprobay)
- Delafloxacin (Baxdela)
- Levofloxacin (Levaquin, Quixin)
- Moxifloxacin (Avelox)
- Not Applicable

In the last year, has your hospital performed any of the following to try to reduce overall fluoroquinolone prescribing? (Check all that apply)

- Tracked rates of fluoroquinolone prescribing
- Antibiotic timeout which includes review of fluoroquinolones
- Discharge intervention de-emphasizing fluoroquinolones
- Specific provider feedback on fluoroquinolone prescribing rates
- Education de-emphasizing fluoroquinolones and suggesting alternative therapy
- Local guidelines recommending against fluoroquinolones AND offering alternatives
- Not Applicable

This is the completion of the survey. Please check your responses for accuracy and select the forward arrow button to submit.

# Stakeholder Surveys

## 2.1 Clinical Vignettes

Next, we will present some case descriptions and ask you questions about antibiotic prescribing decisions. Please answer as best you can, based on the information provided. There are 5 short vignettes.

**Vignette #1:** You have a healthy, 65-year-old woman admitted with community-acquired pneumonia (right upper lobe infiltrate, cough, fever) who is improving well and ready for discharge. She's received three days of ceftriaxone in the hospital.

Would you recommend sending this patient home on an antibiotic?

- Yes
- No

How sure are you about your choice?

- 1 (completely unsure)
- 2
- 3
- 4
- 5
- 6
- 7
- 8
- 9
- 10 (completely sure)

Display This Question: If Would you recommend sending this patient home on an antibiotic? = Yes

What antibiotic would you recommend sending this patient home on?
(Complete slots 2, 3 only if you would recommend more than one antibiotic)

|  | Name of Antibiotic | Days Prescribed at Discharge | How sure are you about this choice? (Please enter a number from 1 to 10, where 1=completely unsure and 10=completely sure. |
| --- | --- | --- | --- |
| Antibiotic #1 |  |  |  |
| Antibiotic #2 |  |  |  |
| Antibiotic #3 |  |  |  |

**Vignette #2** You have a healthy, 65-year-old woman admitted with pneumonia (right upper lobe infiltrate, cough, fever) who had a MRSA pneumonia six months previously. She was recently hospitalized with a STEMI with a successful PCI. This hospitalization, she was unable to produce sputum for analysis. Her MRSA nares is negative, and she is improving well and is ready for discharge. She's received three days of vancomycin and Zosyn in the hospital.

Would you recommend sending this patient home on an antibiotic?

- Yes
- No

How sure are you about your choice?

- 1 (completely unsure)
- 2
- 3
- 4
- 5
- 6
- 7
- 8
- 9
- 10 (completely sure)

Display This Question: If Would you recommend sending this patient home on an antibiotic? = Yes

 What antibiotic would you recommend sending this patient home on?
(Complete slots 2, 3 only if you would recommend more than one antibiotic)

|  | Name of Antibiotic | Days Prescribed at Discharge | How sure are you about this choice? (Please enter a number from 1 to 10, where 1=completely unsure and 10=completely sure. |
| --- | --- | --- | --- |
| Antibiotic #1 |  |  |  |
| Antibiotic #2 |  |  |  |
| Antibiotic #3 |  |  |  |

**Vignette #3** You have a healthy, 65-year-old woman admitted with hypotension, fever, dysuria, and a urine culture growing pan-sensitive e-coli. The patient is improving and ready for discharge. She's received three days of ceftriaxone in the hospital.

Would you recommend sending this patient home on an antibiotic?

- Yes
- No

How sure are you about your choice?

- 1 (completely unsure)
- 2
- 3
- 4
- 5
- 6
- 7
- 8
- 9
- 10 (completely sure)

Display This Question: If Would you recommend sending this patient home on an antibiotic? = Yes

 What antibiotic would you recommend sending this patient home on?
(Complete slots 2, 3 only if you would recommend more than one antibiotic)

|  | Name of Antibiotic | Days Prescribed at Discharge | How sure are you about this choice? (Please enter a number from 1 to 10, where 1=completely unsure and 10=completely sure. |
| --- | --- | --- | --- |
| Antibiotic #1 |  |  |  |
| Antibiotic #2 |  |  |  |
| Antibiotic #3 |  |  |  |

**Vignette #4** You have a healthy, 65-year-old woman with dementia (and no other conditions) admitted with altered mental status. Vital signs remained within normal limits. Her urine culture grew pan-sensitive e-coli. All other work-up has been negative. She was treated with ceftriaxone for three days, IV fluids, and her Ultram was held. She has improved and is ready for discharge. She denies urinary symptoms.

Would you recommend sending this patient home on an antibiotic?

- Yes
- No

How sure are you about your choice?

- 1 (completely unsure)
- 2
- 3
- 4
- 5
- 6
- 7
- 8
- 9
- 10 (completely sure)

Display This Question: If Would you recommend sending this patient home on an antibiotic? = Yes

 What antibiotic would you recommend sending this patient home on?
(Complete slots 2, 3 only if you would recommend more than one antibiotic)

|  | Name of Antibiotic | Days Prescribed at Discharge | How sure are you about this choice? (Please enter a number from 1 to 10, where 1=completely unsure and 10=completely sure. |
| --- | --- | --- | --- |
| Antibiotic #1 |  |  |  |
| Antibiotic #2 |  |  |  |
| Antibiotic #3 |  |  |  |

**Vignette #5** You have a healthy, 65-year-old woman with dementia (and no other conditions) admitted with altered mental status. On admission, her heart rate was 120, blood pressure 80/40, respiratory rate 24, temperature 38.5 C. Her urine culture grew pan-sensitive e-coli. All other work-up has been negative. She was treated with ceftriaxone for three days; IV fluids and her Ultram was held. She has now improved; her vital signs have normalized, and she denies urinary symptoms.

Would you recommend sending this patient home on an antibiotic?

- Yes
- No

How sure are you about your choice?

- 1 (completely unsure)
- 2
- 3
- 4
- 5
- 6
- 7
- 8
- 9
- 10 (completely sure)

Display This Question: If Would you recommend sending this patient home on an antibiotic? = Yes

 What antibiotic would you recommend sending this patient home on?
(Complete slots 2, 3 only if you would recommend more than one antibiotic)

|  | Name of Antibiotic | Days Prescribed at Discharge | How sure are you about this choice? (Please enter a number from 1 to 10, where 1=completely unsure and 10=completely sure. |
| --- | --- | --- | --- |
| Antibiotic #1 |  |  |  |
| Antibiotic #2 |  |  |  |
| Antibiotic #3 |  |  |  |

## 2.2 Pharmacist Survey

The purpose of this study is to learn more about your hospital's approach to antibiotic prescribing. We hope this research will be useful in helping to identify ways to improve antibiotic use. 

The risks of participating in this study are minimal. We will not ask you about sensitive topics, but it is possible that you may feel uncomfortable answering some questions; you may choose not to answer. There is a small risk of loss of confidentiality. All data for this project will be stored on secure computer systems. The information collected in this research will not be used for future studies. 

You may contact the IRB if you have questions regarding your rights as a research participant. Also, contact the IRB if you have questions, complaints or concerns which you do not feel you can discuss with the investigator. The IRB may be reached by phone at …

By completing the information below, you are giving your consent to participate in this research. Thank you for your willingness to participate.

**First, we'd like to ask a few questions about you and your current position.**

What is the formal title for your current position?

________________________________________________________________

Please estimate what percent of your funded effort is spent working with hospitalists (i.e., any inpatient general medicine teams).

________________________________________________________________

How long has it been since you finished terminal training (i.e., fellowship, residency, PharmD)?

- <1 year
- 1 to 2 years
- 2 to 3 years
- 4 to 5 years
- 6 to 7 years
- 8 to 9 years
- 10 years or more

Who is your primary employer?

________________________________________________________________

Do you work at any other hospitals?

- Yes
- No

Display This Question: If Do you work at any other hospitals? = Yes

Please describe your work at other hospitals (where, how often, duties, etc.).

________________________________________________________________

For how many years have you been in your current position?

________________________________________________________________

For how many years have you worked at your current hospital?

________________________________________________________________

**The next questions are about your work environment and responsibilities.**

What are your main duties with the hospital medicine team?

________________________________________________________________

What is your role with antibiotic stewardship?

________________________________________________________________

**For the following questions, please rate how strongly you agree or disagree**

|  | Strongly Disagree | Disagree | Neither Agree nor Disagree | Agree | Strongly Agree |
| --- | --- | --- | --- | --- | --- |
| I am respected by my pharmacist colleagues |  |  |  |  |  |
| I am respected by my hospitalist colleagues |  |  |  |  |  |
| I am considered a valuable member of the clinical team |  |  |  |  |  |

How comfortable are you recommending changes to antibiotic prescriptions if you think guidelines aren't being met?

- Very Uncomfortable
- Uncomfortable
- Neither Comfortable nor Uncomfortable
- Comfortable
- Very Comfortable

For ALL cases in which you review antibiotic prescribing, please estimate the percent of cases you typically believe an alternative treatment would be better (e.g., dose, duration, antibiotic selection).

________________________________________________________________

For ALL cases in which you review antibiotic prescribing, please estimate the percent of cases you actually reach out to the clinician to recommend an antibiotic change (e.g., dose, duration, antibiotic selection).

________________________________________________________________

When you recommend a change to an antibiotic prescription, please estimate the percent of the time your recommendations are adopted.

________________________________________________________________

At your hospital, are there specific pharmacists who specialize in transitions of care?

- Yes
- No

Display This Question:

If At your hospital, are there specific pharmacists who specialize in transitions of care? = Yes

What do pharmacists who specialize in transitions of care do, with respect to discharge?

________________________________________________________________

Display This Question:

If At your hospital, are there specific pharmacists who specialize in transitions of care? = Yes

How do you interact with pharmacists who specialize in transitions of care?

________________________________________________________________

Display This Question:

If At your hospital, are there specific pharmacists who specialize in transitions of care? = Yes

How could the interaction between medicine clinical pharmacists and discharge pharmacists be improved?

________________________________________________________________

________________________________________________________________

What is your role in the discharge process?

________________________________________________________________

What is your reporting structure, i.e., who do you report to?

________________________________________________________________

How important do you think antibiotic stewardship is?

- 1 (very unimportant)
- 2 (unimportant)
- 3 (neutral)
- 4 (important)
- 5 (very important)

Does your hospital have a "meds to bed" program?

- Yes
- No

Display This Question: If Does your hospital have a "meds to bed" program? = Yes

Are antibiotics included in the meds to bed program? Please describe.

________________________________________________________________

Finally, please complete the following demographic questions.

Name (this information will be removed from the final dataset to protect your anonymity).

________________________________________________________________

Gender

- Male
- Female
- Non-binary / third gender
- Prefer Not to Disclose

Race

- American Indian or Native Alaskan
- Asian
- Black or African American
- Native Hawaiian or Other Pacific Islander
- White or Caucasian
- Other __________________________________________________
- Prefer Not to Disclose

Ethnicity

- Hispanic or Latino
- Non-Hispanic or Latino
- Prefer Not to Disclose

## Hospitalist Survey

The purpose of this study is to learn more about your hospital's approach to antibiotic prescribing. We hope this research will be useful in helping to identify ways to improve antibiotic use. 

The risks of participating in this study are minimal. We will not ask you about sensitive topics, but it is possible that you may feel uncomfortable answering some questions; you may choose not to answer. There is a small risk of loss of confidentiality. All data for this project will be stored on secure computer systems. The information collected in this research will not be used for future studies. 

You may contact the IRB if you have questions regarding your rights as a research participant. Also, contact the IRB if you have questions, complaints or concerns which you do not feel you can discuss with the investigator. The IRB may be reached by phone at …

By completing the information below, you are giving your consent to participate in this research. Thank you for your willingness to participate.

**First, we'd like to ask a few questions about you and your current position.**

What is the formal title for your current position?

________________________________________________________________

Please estimate what percent of your funded effort is devoted to patient care.

________________________________________________________________

How long has it been since you finished terminal training (i.e., fellowship, residency, other degree)

- <1 year
- 1 to 2 years
- 2 to 3 years
- 4 to 5 years
- 6 to 7 years
- 8 to 9 years
- 10 years or more

Who is your primary employer?

________________________________________________________________

Do you work at any other hospitals?

- Yes
- No

Display This Question: If Do you work at any other hospitals? = Yes

Please describe your work at other hospitals (where, how often, duties, etc.).

________________________________________________________________

For how many years have you been in your current position?

________________________________________________________________

For how many years have you worked at your current hospital?

________________________________________________________________

**The next questions are about your work environment and responsibilities.**

Do hospitalists at your hospital work at multiple hospitals?

- Yes
- No
- Don't Know

Approximately how many hospitalists work at your hospital?

________________________________________________________________

How many hospitalist teams are there?

________________________________________________________________

On an average shift, how many patients do you care for?

________________________________________________________________

What types of patients do you care for? (check all that apply)

- General medical patients
- Surgery patients
- Patients in the ICU
- Patients in the ED
- Other (specify) __________________________________________________

How do hospitalists engage with clinical pharmacists at your hospital?

________________________________________________________________

Do you work with Advanced Practice Professionals/ Clinicians (APPs/ APCs)? (i.e., Nurse Practitioners, Physician Assistants)

- Yes
- No

Display This Question: If Do you work with Advanced Practice Professionals/ Clinicians (APPs/ APCs)? (i.e., Nurse Practition... = Yes

Please describe what role APPs/ APCs play with your hospital medicine group and how you work with them.

________________________________________________________________

________________________________________________________________

________________________________________________________________

________________________________________________________________

________________________________________________________________

For the following questions, please rate how strongly you agree or disagree

|  | (1) Strongly Disagree | (2) Disagree | (3) Neither Agree nor Disagree | (4) Agree | (5) Strongly Agree |
| --- | --- | --- | --- | --- | --- |
| I am respected by my colleagues |  |  |  |  |  |
| Clinical pharmacists are valuable members of the clinical team |  |  |  |  |  |
| Clinical pharmacists are knowledgeable about antibiotic use |  |  |  |  |  |

When clinical pharmacists suggest changes to antibiotics you have planned/ prescribed, what percent of the time do you agree with their assessment and make the suggested change? (estimate)

________________________________________________________________

How important do you think antibiotic stewardship is?

- 1 (very unimportant)
- 2 (unimportant)
- 3 (neutral)
- 4 (important)
- 5 (very important)

Do you have any financial incentive structure that potentially allows you to receive more than your base salary?

- Yes
- No

Display This Question: If Do you have any financial incentive structure that potentially allows you to receive more than yo... = Yes

What is the incentive structure?

________________________________________________________________

________________________________________________________________

________________________________________________________________

________________________________________________________________

________________________________________________________________

Display This Question: If Do you have any financial incentive structure that potentially allows you to receive more than yo... = Yes

Are quality metrics part of the incentive/bonus?

- Yes
- No

Display This Question: If Are quality metrics part of the incentive/bonus? = Yes

Please describe these quality metrics.

________________________________________________________________

________________________________________________________________

________________________________________________________________

Display This Question: If Do you have any financial incentive structure that potentially allows you to receive more than yo... = Yes

How and by whom is the incentive structure determined?

________________________________________________________________

________________________________________________________________

________________________________________________________________

**Finally, please complete the following demographic questions.**

Name (this information will be removed from the final dataset to protect your anonymity).

________________________________________________________________

Gender

- Male
- Female
- Non-binary / third gender
- Prefer Not to Disclose

Race

- American Indian or Native Alaskan
- Asian
- Black or African American
- Native Hawaiian or Other Pacific Islander
- White or Caucasian
- Other __________________________________________________
- Prefer Not to Disclose

Ethnicity

- Hispanic or Latino
- Non-Hispanic or Latino
- Prefer Not to Disclose

## 2.4 Antibiotic Stewardship Leader Survey

The purpose of this study is to learn more about your hospital's approach to antibiotic prescribing. We hope this research will be useful in helping to identify ways to improve antibiotic use. 

The risks of participating in this study are minimal. We will not ask you about sensitive topics, but it is possible that you may feel uncomfortable answering some questions; you may choose not to answer. There is a small risk of loss of confidentiality. All data for this project will be stored on secure computer systems. The information collected in this research will not be used for future studies. 

You may contact the IRB if you have questions regarding your rights as a research participant. Also, contact the IRB if you have questions, complaints or concerns which you do not feel you can discuss with the investigator. The IRB may be reached by phone at …

By completing the information below, you are giving your consent to participate in this research. Thank you for your willingness to participate.

**First, we'd like to ask a few questions about you and your current position.**

What is the formal title for your current position?

________________________________________________________________

Please estimate what percent of your funded effort is spent working on antibiotic stewardship.

________________________________________________________________

How long has it been since you finished terminal training (i.e., fellowship, residency, PharmD)?

- <1 year
- 1 to 2 years
- 2 to 3 years
- 4 to 5 years
- 6 to 7 years
- 8 to 9 years
- 10 years or more

Who is your primary employer?

________________________________________________________________

Do you work at any other hospitals?

- Yes
- No

Display This Question: If Do you work at any other hospitals? = Yes

Please describe your work at other hospitals (where, how often, duties, etc.).

________________________________________________________________

For how many years have you been in your current position?

________________________________________________________________

For how many years have you worked at your current hospital?

________________________________________________________________

**The next questions are about your work responsibilities and antibiotic stewardship program.**

Please describe your other roles besides antibiotic stewardship (if no other responsibilities, please type "none").

________________________________________________________________

________________________________________________________________

Approximately when was your antibiotic stewardship program created?

________________________________________________________________

Who leads your antibiotic stewardship program, and what are their qualifications?

________________________________________________________________

Who participates on your antibiotic stewardship team (i.e., what roles are represented, e.g., ID physicians, hospitalists, administrators, etc.)?

________________________________________________________________

________________________________________________________________

________________________________________________________________

Who do you wish participated on your antibiotic stewardship team (in addition to those already participating)?

________________________________________________________________

How frequently does your antibiotic stewardship team meet (include frequency for subgroup meetings, if any)?

________________________________________________________________

Do members of the antibiotic stewardship team regularly attend the meetings? Please describe.

________________________________________________________________

Do clinical/medicine pharmacists play a role in antibiotic stewardship? Please describe.

________________________________________________________________

Do ID pharmacists play a role in antibiotic stewardship? Please describe.

________________________________________________________________

What is the reporting structure for the antibiotic stewardship team (i.e., who does the stewardship program report to)?

________________________________________________________________

How important do you think antibiotic stewardship is?

- 1 (very unimportant)
- 2 (unimportant)
- 3 (neutral)
- 4 (important)
- 5 (very important)

**Finally, please complete the following demographic questions.**

Name (this information will be removed from the final dataset to protect your anonymity).

________________________________________________________________

Gender

- Male
- Female
- Non-binary / third gender
- Prefer Not to Disclose

Race

- American Indian or Native Alaskan
- Asian
- Black or African American
- Native Hawaiian or Other Pacific Islander
- White or Caucasian
- Other __________________________________________________
- Prefer Not to Disclose

Ethnicity

- Hispanic or Latino
- Non-Hispanic or Latino
- Prefer Not to Disclose

## 2.5 Hospital Leadership Survey

The purpose of this study is to learn more about your hospital's approach to antibiotic prescribing. We hope this research will be useful in helping to identify ways to improve antibiotic use. 

The risks of participating in this study are minimal. We will not ask you about sensitive topics, but it is possible that you may feel uncomfortable answering some questions; you may choose not to answer. There is a small risk of loss of confidentiality. All data for this project will be stored on secure computer systems. The information collected in this research will not be used for future studies. 

You may contact the IRB if you have questions regarding your rights as a research participant. Also, contact the IRB if you have questions, complaints or concerns which you do not feel you can discuss with the investigator. The IRB may be reached by phone at …

By completing the information below, you are giving your consent to participate in this research. Thank you for your willingness to participate.

**First, we'd like to ask a few questions about you and your current position.**

What is the formal title for your current position?

________________________________________________________________

Please estimate what percent of your funded effort is spent working on antibiotic stewardship.

________________________________________________________________

How long has it been since you finished terminal training (i.e., fellowship, residency, PharmD)?

- <1 year
- 1 to 2 years
- 2 to 3 years
- 4 to 5 years
- 6 to 7 years
- 8 to 9 years
- 10 years or more

Who is your primary employer?

________________________________________________________________

Do you work at any other hospitals?

- Yes
- No

Display This Question: If Do you work at any other hospitals? = Yes

Please describe your work at other hospitals (where, how often, duties, etc.).

________________________________________________________________

For how many years have you been in your current position?

________________________________________________________________

For how many years have you worked at your current hospital?

________________________________________________________________

How important do you think antibiotic stewardship is?

- 1 (very unimportant)
- 2 (unimportant)
- 3 (neutral)
- 4 (important)
- 5 (very important)

**Finally, please complete the following demographic questions.**

Name (this information will be removed from the final dataset to protect your anonymity).

________________________________________________________________

Gender

- Male
- Female
- Non-binary / third gender
- Prefer Not to Disclose

Race

- American Indian or Native Alaskan
- Asian
- Black or African American
- Native Hawaiian or Other Pacific Islander
- White or Caucasian
- Other __________________________________________________
- Prefer Not to Disclose

Ethnicity

- Hispanic or Latino
- Non-Hispanic or Latino
- Prefer Not to Disclose

# Interview Guides

## 3.1 Pharmacist Interview Guide

Good afternoon [or morning]. Thanks for joining us today. **introduce team members**

For the next hour, our plan is to have a conversation with you about antibiotic prescribing. The goal of the study is to identify ways to improve antibiotic use. We will be recording this zoom meeting today but will de-identify all of the transcripts to protect your identity and data will only be shared in aggregate for research purposes. If at any point you want to stop or skip any questions you are free to do so.

I really want to thank you again for participating!

[questions added ad hoc by site based on discussions with others]

**Actual Interview Questions**

1. To start off, can you briefly describe your role and position at [your hospital]?
   1. Probes
      1. Same service/handoffs?
      2. How split. Geographic?
      3. Plan for when new medicine teams are added?
2. What is your role(s) in antibiotic stewardship?
   1. Probes
      1. Different weekend vs. weekday?
      2. What does that involve?
3. What’s your relationship like with the stewardship program?
   1. Probes
      1. Do you consider yourself to be part of the antibiotic stewardship team?
         1. Would the stewardship team agree with that?
      2. Who do you interact with from the stewardship team?
      3. Do you attend meetings of the antibiotic stewardship team?
      4. How’s the relationship with [your stewardship leader]?
      5. Any additional help you’d like from your stewardship program?
      6. How do they get your input?
      7. Can you ask questions of stewardship team during day-to-day interactions?
         1. How does that work?
4. How would you describe your relationship with the hospitalists?
   1. What rounding style do you find most conductive to including you in care?
   2. Probes
      1. How do you interact with the hospitalist teams?
5. Let’s say you have a patient who’s being discharged who is currently on antibiotics. Walk me through the process for deciding what antibiotics are prescribed at discharge?
   1. Probes
      1. How find out who is being discharged? Notifications?
      2. Is this a standardized process or is there a lot of variation?
      3. Are there other medications with a standard process?
         1. Why those medications?
      4. How are you involved in this process?
         1. How do you identify patients that are expected to be discharged?
         2. How do you identify patients that are expected to be discharged on oral antibiotics?
         3. Can you make changes to medications ordered by physicians? If so, how is this communicated to clinician? Documented?
         4. Are you involved (another pharmacist?) in the discharge medication reconciliation process?
6. Please describe what process, if any, you have to check for the appropriateness of antibiotics before discharge?
   1. Probes
      1. What (if any) guidelines or tools do you use to decide antibiotic selection?
      2. Do you ever discuss antibiotics with the clinician before discharge?
         1. Do you have a routine process for having this conversation?
      3. What happens if you don’t agree with a clinician’s antibiotic selection? Are you able to push back or discuss the clinician’s antibiotic selection? Please describe that process.
         1. How do you communicate recommendations for changes?
         2. How do the physicians respond to those conversations?
   2. Can you make changes to medications ordered by physicians? If so, how is this communicated to clinician? Documented?
   3. Are you involved (another pharmacist?) in the discharge medication reconciliation process?
7. Describe the handoff process between pharmacists.
   1. Probe: Is antibiotic information included?
8. Is there a TOC pharmacist?
   1. Probes
      1. If yes, describe the relationship between inpatient and TOC pharmacy
      2. How long have they been around
      3. Communication, handoffs
      4. How could it improve?
9. In what situations have you noticed clinicians prescribing unnecessary antibiotics?
   1. Probes: (How about unnecessary antibiotic prescribing by hospitalists or general internists?)
10. I’d like to discuss antibiotic treatment of asymptomatic bacteriuria. Do you find that clinicians over-prescribe antibiotics for ASB?
    1. Probes
       1. Have you noticed any reasons for continued prescribing?
       2. How do you approach clinicians who prescribe for these patients?
       3. Does your hospital have an initiative in this area? Please describe.
11. In what situations have you noticed that clinicians prescribe antibiotics for longer than needed?
    1. Probes
       1. How do you approach this situation?
       2. How is appropriate duration currently determined?
12. I’d like to discuss antibiotic duration in patients with CAP. Do you find that clinicians prescribe antibiotics for longer than 5 days for CAP patients?
    1. Probes
       1. What are some reasons for longer prescriptions? (HCAP???)
       2. How do you approach clinicians who prescribe longer durations for these patients?
       3. Does your hospital have an initiative in this area? Please describe.
13. Are there any situations where you’ve noticed clinicians might prescribe fluoroquinolone antibiotics when an alternative is preferred?
    1. Probes: How do you approach this situation?
14. What’s the process for educating patients about the antibiotics they’re being sent home on?
    1. Probes
       1. What is your role in this process?
       2. Do you discuss potential side effects with patient?
       3. What if the patient is confused or has difficulty understanding the information?
15. What’s the process for ensuring a patient gets their antibiotic at discharge?
    1. Probes
       1. How does the outpatient pharmacy receive information about the antibiotics?
       2. Does your hospital have a “meds to bed” program?
       3. What is the role of pharmacy?
16. Describe the process, if any, for following up with patients who are discharged on antibiotics?
    1. Probes: How do you find out if a patient had an adverse event from their antibiotic?
17. What current intervention(s) exist to improve sepsis care at your hospital.
    1. Probes: How do these initiatives affect antibiotic prescribing?
18. Are you aware of any current or prior intervention(s) exist to improve prescribing at discharge?
    1. Probes:
       1. What worked well? What didn’t work well?
       2. What are the biggest barriers you’d anticipate to such an intervention?
       3. Please describe any interventions you anticipate your hospital will initiate in the future?
       4. If you could design an intervention to improve discharge prescribing, what would it be?
19. Do you ever receive feedback about your work?
    1. Probes
       1. From whom?
       2. What did the feedback contain?
       3. If yes, did you find it helpful?
       4. If no, would you like to receive feedback? What type?
       5. Does the feedback ever include information on antibiotic prescribing? Prescribing specifically at discharge?
          1. If no, do you think it would be valuable to know those data?
       6. Did you change any of your practices based on the feedback?
20. I want to start broadly. How would you describe your hospitals culture?
21. Going back to our discussion of hospitalists, what do you do if they disagree with your recommendation?
    1. How common is that?
    2. Do you have someone you can escalate issues to?
22. (only ask women); What role (if any) do you think gender has to play in whether your recommendations are considered.
23. Generally speaking, what do you think are the biggest priorities of your hospital’s leadership?
24. Prompt: How are hospital priorities set? By whom? Role of external metrics/agencies?
25. Before I show you your hospitals prescribing rates, I’d like to know how you think your hospital performs compared to other hospitals regarding antibiotic prescribing at discharge?
26. (show them their prescribing rates) Why do you think your hospital is doing how it is compared to other hospitals?
27. What additional information would you like to see in your hospital’s antibiotic prescribing guidelines?
28. How did you learn your current practices?
    1. What other information sources do you use to stay up to date on antibiotic prescribing?
29. Finally, if you were to give another hospital advice about improving appropriate antibiotic use at discharge, what would you tell them to do?

Is there anything else you would like to add?

That is the end of the questions that we have for you. Do you have any questions for me or any other comments on anything that we have discussed today? Thank you very much for taking the time to participate in this interview.

## 3.2 Hospitalist Interview Guide

Good afternoon [morning]. Thanks for joining us today. **introduce team members**

For the next hour, our plan is to have a conversation with you about antibiotic prescribing. Over the next two weeks we’ll be meeting with hospitalists, pharmacists, and stewards about practices related to antibiotic prescribing. The study goal is two-fold. First, at the end of the visit we’ll provide a summary of things going really well at [your hospital] as well as potential ways to improve. Then, since we’re working with a group of hospitals, if there’s something we think other hospitals could learn from what [your hospital] is doing, we’d potentially share that with other hospitals as well. We will be recording this zoom meeting today but will de-identify all of the transcripts to protect your identity, and data will only be shared in aggregate for research purposes. If at any point you want to stop or skip any questions - or take the 5^th^! - you are free to do so.

I really want to thank you again for participating! Do you have any questions before we get started?

**Actual Interview Questions**

1. To start off, can you briefly describe your role and position at [your hospital]?
   1. Can you tell me a bit about the structure and role of APPs with hospital medicine?
2. On a day-to-day basis, how do you interact with clinical pharmacists? What about transitions of care pharmacists?
   1. Probes
      1. How would you describe the relationship between hospitalists and clinical pharmacists?
      2. How do you feel about pharmacist feedback
      3. Is the information from pharmacists consist?
      4. Is there a difference in pharmacist approach?
      5. What is your preferred way of being approached?
3. What is the role of the antibiotic stewardship team at your hospital? (initiatives)
   1. Probes
      1. What kind of ways do you interact?
      2. What would you say are the top priorities related to stewardship?
4. On a day-to-day basis, how do you interact with ID physicians?
   1. Probes
      1. Are they available for onsite/virtual/phone consultation?
      2. What’s your relationship with ID physicians here?
      3. What is their role in antibiotic stewardship?
5. I want you to imagine you have a patient who’s being discharged from your service who is currently on antibiotics. Can you walk me through your decision-making process for what you prescribe at discharge?
   1. Probes
      1. What (if any) guidelines or tools do you use to decide antibiotic selection? Hospital guidelines? System/sound group?
      2. What clinical information do you use to make this decision?
      3. Do you consult with other colleagues (e.g., pharmacists, ID physicians) when deciding? (if yes) Who? Format? Weekends?
6. What order-sets or decision-support mechanisms exist in your EHR related to antibiotics?
   1. Probes
      1. Do you use these?
   2. (If there are no tools in the EHR) Do you think having some type of tool in the EHR would be helpful? Please explain
   3. Please describe any information you wish was available in EHR?
7. Now let’s say you have a similar scenario with a patient ready to be discharged on antibiotics. But in this case, it’s your first day on service and **you’re taking over for a colleague**. How does that affect your decision-making process for what you prescribe at discharge?
   1. Probes
      1. Describe the handoff process between hospitalists.
      2. Is antibiotic information included?
      3. Do you ever change the recommendation left to you by a colleague? If yes, why?
8. How do you decide how long a patient needs antibiotics when they’re discharged?
   1. What do you do if you’re unsure about how long a patient needs antibiotics?
9. What is the typical duration of antibiotics that you prescribe to patients diagnosed with CAP?
   1. Probes
      1. What might make that longer?
      2. How has your practice for duration of therapy for antibiotics changed over time?
10. What led to the change(s)?

1. Medicine has a lot of gray areas. What about patients who have unclear chest x-ray findings or symptoms. How do you decide whether to treat for community-acquired pneumonia?
2. Probe – Has your practice changed over time?
3. Describe your perspective on prescribing fluoroquinolones to patients?
4. Probes
5. How did you come about this perspective?
6. Has your practice changed over time?
7. Describe your practice around antibiotic de-escalation.
   1. Probes
8. How did you come about this practice?
9. Has your practice changed over time?
10. How do you handle antibiotic prescribing in patients with asymptomatic bacteriuria?
    1. Probes
       1. Has your practice changed over time? What changed? What led to the change(s)?
       2. How do you handle patients who are altered and can’t tell you about symptoms?
       3. Has your practice changed over time? What changed? What led to the change(s)?
11. What current intervention(s) exist to improve antibiotic use at your hospital.
12. How do these initiatives affect antibiotic prescribing?
13. What current intervention(s) exist to improve sepsis care at your hospital.
14. How do these initiatives affect antibiotic prescribing?

1. What expectations do patients (or their families) have about antibiotics at discharge?
2. Describe the process for educating patients about the antibiotics they’re being sent home on.
   1. Probes:
      1. What is your role in this process?
      2. Do you discuss potential side effects with patient?
3. Describe the process for communicating with the patient’s PCP about the antibiotics they’re being sent home on.
4. How do you find out if a patient had an adverse event from their antibiotic?
5. Do you ever receive any feedback reports or data on antibiotic prescribing practices?
6. Probes
7. If yes, What did the feedback contain? Do you find those helpful? Did you change the way you prescribe at all based on the feedback?
8. If no, do you think it would be valuable to know those data?
9. I want to start broadly. How would you describe your hospital’s culture? (*fit this question is where it flows; moved order midway through site Visit #1)*
10. Generally speaking, what do you think are the biggest priorities of your hospital’s leadership?
    1. Prompt:
       1. How are hospital priorities set? By whom?
       2. Role of external metrics/agencies?
11. Before I show you your hospitals prescribing rates, I’d like to know how you think your hospital performs compared to other hospitals regarding antibiotic prescribing at discharge.

1. (show them their prescribing rates) Why do you think your hospital is doing how it is compared to other hospitals?
2. Prompt: Are there things you feel your hospital could be doing to reduce antibiotic overuse?
3. Please describe any additional information you’d like to see in your hospital’s antibiotic guidelines.
   1. Any feedback you’d like to give to the antibiotic stewardship team?
4. Finally, if you were to give another hospital advice about improving appropriate antibiotic use at discharge, what would you tell them to do? (Organization)
5. Is there anything else you would like to add?

That is the end of the questions that we have for you. Now do you have any questions for me or any other comments on anything that we have discussed today? **Thank you very much for taking the time to participate in this interview**. We really appreciate your time.

## Antibiotic Stewardship Leader Interview Guide

Good afternoon [morning]. Thanks for joining us today. **introduce team members**

For the next hour, our plan is to have a conversation with you about antibiotic prescribing. The goal of the study is to identify ways to improve antibiotic use. We will be recording this zoom meeting today but will de-identify all of the transcripts to protect your identity, and data will only be shared in aggregate for research purposes. If at any point you want to stop or skip any questions you are free to do so.

I really want to thank you again for participating!

1. We would like to start by better understanding everyone’s role. Would you please tell us a bit about your responsibilities as part of the stewardship team?
2. Can you describe your antibiotic stewardship team?
   1. Probes:
      1. ASP committee: When established? How often meet? Who report to?
      2. How is it structured?
      3. Who is involved?
      4. What are the main current activities?
3. What is the role of ID pharmacists in antibiotic stewardship?
   1. Probes:
      1. How do you feel the clinical pharmacists react to antibiotic stewardship initiatives?
      2. Anything you wish they could do better?
4. What is the role of clinical pharmacists in antibiotic stewardship?
   1. What about discharge/TOC pharmacists?
   2. Probes:
      1. How do you feel the clinical pharmacists react to antibiotic stewardship initiatives?
      2. Anything you wish they could do better?
      3. Difficulty between ID pharmacist and clinical pharmacists?
5. What is the role of ID physicians in antibiotic stewardship?
   1. Probes:
      1. How do you feel the clinical pharmacists react to antibiotic stewardship initiatives?
      2. Anything you wish they could do better?
6. What institutional support do you have for stewardship?
   1. Probes:
      1. Dedicated effort, FTE, money, IT support, pharmacy
7. What additional support do you wish you had?
   1. Probes: IT support
8. Please describe your IT support?
   1. Probes: Barriers? Ordersets?
9. Please describe the top antibiotic stewardship priorities of your hospital leadership?
   1. Probes:
      1. What determines these priorities?
      2. What is the role of external metrics/agencies in setting priorities?
      3. NHSN
10. What are YOUR top stewardship priorities?
11. How does the stewardship team set its priorities?
    1. Probes:
       1. Leadership
       2. External metrics
       3. Data-based
       4. NHSN
12. Describe your relationship with [your health system]. How does it affect antibiotic stewardship? (or if not part of system) Are you involved in any stewardship activities with other hospitals?
13. How would you describe [your hospital’s] culture around antibiotic stewardship?
    - - 1. How did it come to be this way?
14. Describe your process for making antibiotic stewardship guidelines?
    1. Probes
       1. How do you arrive at consensus?
       2. Who has to approve or review guidelines before they’re published?
       3. What’s the process for keeping them up to date? How often are they reviewed?
       4. What’s the process for disseminating them? Incorporating them into existing workflows/ordersets?
15. Describe your relationship with the hospital medicine group**s** at [your hospital]
    1. Probes
       1. Does stewardship have a hospitalist champion?
       2. How do you feel hospitalists react to antibiotic stewardship initiatives?
       3. Anything you wish they could do better?
       4. What could be done to make that happen?
16. Describe your relationship with infection control.
    1. Probes
       1. Are you on their committees?
       2. Are they on your committees?
       3. How do you make sure you don’t work duplicate work?
17. What is the relationship between antibiotic stewardship and sepsis improvement initiatives?
    1. Probes
       1. Are you on their committees?
       2. Are they on your committees?
       3. What do you do if there’s disagreement?
18. Tell me about what data you use to guide antibiotic stewardship initiatives?
    1. Probes
       1. Is there any additional data you wish you had access to?
19. Do you ever report or provide feedback data to providers on antibiotic prescribing practices?
    1. Probes
       1. If no, what prevents this?
       2. If yes, What did the feedback contain? Did it contain any information on antibiotic use at discharge? Do you think these reports were helpful? Have they caused providers to change their antibiotic prescribing behaviors? What could improve the feedback?
20. Does your facility collect data on antibiotic prescribing at discharge?
    1. Probes:
       1. If no, what prevents this? Would it be helpful?
       2. If yes, how is this collected?
       3. If yes, is this prospective/retrospective?
21. Have you provided any education to providers on antibiotic prescribing? (Provider/Internal Environment)
    1. If yes, what did the education entail? did it include instructions or details on prescribing at discharge? who provided that education? did it have any effect on prescribing practices? Why/why not?
22. Do you collaborate with any external partners (e.g., CDC, research projects, collaboratives?)
    1. Probes
       1. If yes, please describe. (how long, what do, data?)
       2. If no, what prevents this?
23. Do you have any diagnostic stewardship initiatives?
    1. Probes
       1. If yes, please describe.
       2. If no, what prevents this?
       3. How would you describe the culture around diagnostic testing?
          1. How did it come to be this way?
       4. Do you have any diagnostic stewardship interventions for UTI/ASB?
24. Have there been any interventions (now or prior) to improve antibiotic use for pneumonia? Please describe.
    1. Probes
       1. What worked well? What didn’t work well?
       2. How do you think use of antibiotics in pneumonia patients could be improved at your hospital?
       3. Why do you think some clinicians still prescribe for than 5-days?
       4. How would you operationalize this change?
       5. What barriers, if any, do you foresee in making this happen?
       6. Is this a priority for you/ASP/clinicians/your hospital?
25. Have there been any interventions (now or prior) to improve antibiotic use for UTI? Please describe.
    1. Probes
       1. What worked well? What didn’t work well?
       2. At your hospital, how do you think use of antibiotics in UTI patients could be improved?
       3. How would you operationalize this change?
       4. What barriers do you foresee in making this happen?
       5. Is this a priority for you/ASP/clinicians/your hospital?
26. Have there been any interventions (now or prior) to improve fluoroquinolone use? Please describe.
    1. Probes:
       1. What worked well? What didn’t work well?
       2. How would you operationalize this change?
       3. What barriers do you foresee in making this happen?
27. Have you had any interventions to improve antibiotic use at discharge?
    1. Probes
       1. If yes, what worked well? What didn’t work well?
       2. If no, are there any interventions planned for the future?
       3. What do you think is the biggest opportunity for improvement in discharge antibiotic use at this hospital? (Specific type of patient? Specific provider? Specific type of antibiotic?)
28. What do you think would be the best way to improve discharge antibiotic use at this hospital?
    1. Probes
       1. What would you need to make that happen?
       2. How would you operationalize this change?
       3. What barriers do you foresee in making this happen?
29. Describe the process for educating patients about the antibiotics they’re being sent home on?
    1. Probes
       1. What is the role of stewardship in this process?
       2. Is there information about potential side effects?
          1. Is there a process to help patients who are confused or have difficulty understanding the information.
       3. Were there any external metrics that influenced this practice?
30. Does the antibiotic stewardship program receive feedback on how it’s doing?
    1. Probes
       1. If yes, what did the feedback contain? from whom? is the feedback helpful? did you change any stewardship activities based on the feedback?
       2. If no, do you think it would be valuable to receive feedback? What type of feedback would you like to receive?
31. Before I show you your hospitals prescribing rates, I’d like to know how you think your hospital performs compared to other hospitals in regard to antibiotic prescribing at discharge.
32. (show them their prescribing rates) I’d love to hear your reflections on what you’ve seen
    1. Probes:
       1. How do you think your hospital could improve appropriate antibiotic use?
       2. What barriers would you anticipate?
33. (if woman) What role do you think gender has played in the way your recommendations are perceived as a steward?
34. How did you learn your current practices?
    1. What information sources do you use to stay up to date on antibiotic prescribing?
35. Finally, if you were to give another hospital advice about improving appropriate antibiotic use at discharge, what would you tell them to do?
36. What information would you like us to collect from pharmacists and hospitalists here?

Is there anything else you would like to add?

That is the end of the questions that we have for you. We know this took a lot of your time and you’re very busy. We really appreciate you taking the time to speak with us today. Before we end, do you have any questions for me or any other comments on anything that we have discussed today?

## 3.4 Hospital Leadership Interview Guide

Good afternoon. Thanks for joining us today. **introduce team members**

For the next hour, our plan is to have a conversation with you about antibiotic prescribing. The goal of the study is to identify ways to improve antibiotic use. We will be recording this zoom meeting today but will de-identify all of the transcripts to protect your identity, and data will only be shared in aggregate for research purposes. If at any point you want to stop or skip any questions you are free to do so.

I really want to thank you again for participating!

1. We would like to start by better understanding everyone’s role. Could you to please tell us a bit about yourself and your responsibilities.
   1. Probes:
      1. What is your formal title?
      2. How long have you worked at your hospital? How long have you been in your current position?
      3. Do you work at any other hospitals?
      4. Who’s your primary employer?
2. Tell me about the structure of your quality/leadership team?
   1. Probes:
      1. (When was it created?)
      2. What’s the reporting structure? Who leads the program?
      3. Who participates in this team? (main team? Broad team?)
         1. Who else do you wish participated?
         2. (Antibiotic stewardship, pharmacists, hospitalists)
      4. How frequently does this team meet?
         1. Do the members of the team attend meetings regularly?
3. What institutional support do you have (dedicated effort, FTE, money, IT support)?
4. What additional support do you wish you had?
5. What IT support do you have?
   1. Probes: order sets, decision-support, data gather
6. Describe your relationship with [your health system]. How does it affect antibiotic stewardship? [Only asked for hospitals part of larger system]
   1. Probes:
      1. Are you allowed to implement local changes without system approval?
7. How would you describe the culture around quality and patient safety?
   1. Probes: How did it come to be this way?
8. What are your hospital’s (department) top quality priorities?
   1. Probes:
      1. How are those priorities determined?
      2. What is the role of external metrics or agencies in determining quality priorities?
9. How do you obtain data to guide institutional priorities?
   1. Probes
      1. What resources are available for obtaining that data?
      2. Is there any additional data you wish you had?
10. What data have you seen on antibiotic use at your hospital?
11. What are the top antibiotic stewardship priorities of your hospital’s leadership?
    1. Probes
       1. What are YOUR top stewardship priorities?
       2. Describe your relationship with the antibiotic stewardship team
12. Describe your process for making guidelines?
    1. Probes
       1. How do you arrive at consensus?
       2. Who has to approve or review guidelines before they’re published?
       3. What’s the process for keeping them up to date?
       4. What’s the process for disseminating them? Incorporating them into existing workflows/ordersets?
13. Describe your relationship with the clinical/medicine pharmacists here.
    1. Probe
       1. ID pharmacists?
       2. What is their role in quality?
14. Describe your relationship with the hospital medicine group here.
    1. Probe
       1. What is their role in quality?
15. Describe your relationship with the antibiotic stewardship program here. (Ali Earl)
    1. probe
       1. What is their role in quality?
       2. How do you feel the stewardship team is doing in terms of antibiotic stewardship?
          1. Anything you wish they could do better?
          2. What could be done to make that happen?
16. How does the stewardship program receive feedback on how it’s doing?
    1. Probes:
       1. What did the feedback contain?
       2. From who?
17. If someone wanted to initiate a quality improvement protocol at your hospital, what would the process look like?
    1. Probes:
       1. Who would need to approve?
       2. What data?
       3. What outcomes?
       4. How obtain resources?
       5. How decide what to move forward on?
18. (only ask women); “I’m not sure exactly how to answer this question but what I’ve heard….What role (if any) do you think gender has to play in whether your recommendations are considered.
19. Before I show you your hospital’s prescribing rates, I’d like to know how you think this institution performs compared to other hospitals in regard to antibiotic prescribing at discharge.
20. (show them their prescribing rates) Why do you think your hospital is doing better than others/not as well as others?
    1. Probes: For those that are not doing well
       1. How do you think your hospital could improve appropriate antibiotic use?
       2. Any specific areas of improvement?
       3. What barriers would you anticipate?
       4. What’s the one thing that could help the most?
    2. Probes: For those that are doing well
       1. To what do you attribute your levels of appropriate antibiotic use? Why are you so successful?
       2. Are there any areas that you would like to improve?
       3. How could that be achieved?
21. What do you think would be the best way to improve discharge antibiotic use at this hospital? (Tools and Tech)
    1. Probes
       1. What would you need to make that happen?
22. Finally, if you were to give another hospital advice about improving appropriate antibiotic use at discharge, what would you tell them to do? (Organization)

Is there anything else you would like to add?

That is the end of the questions that we have for you. Now do you have any questions for me or any other comments on anything that we have discussed today? Thank you very much for taking the time to participate in this interview.

# Antibiotic Overuse

## 4.1 eFigure 1. Antibiotic Overuse at Hospital Discharge – Community-Acquired Pneumonia and Urinary Tract Infection Combined


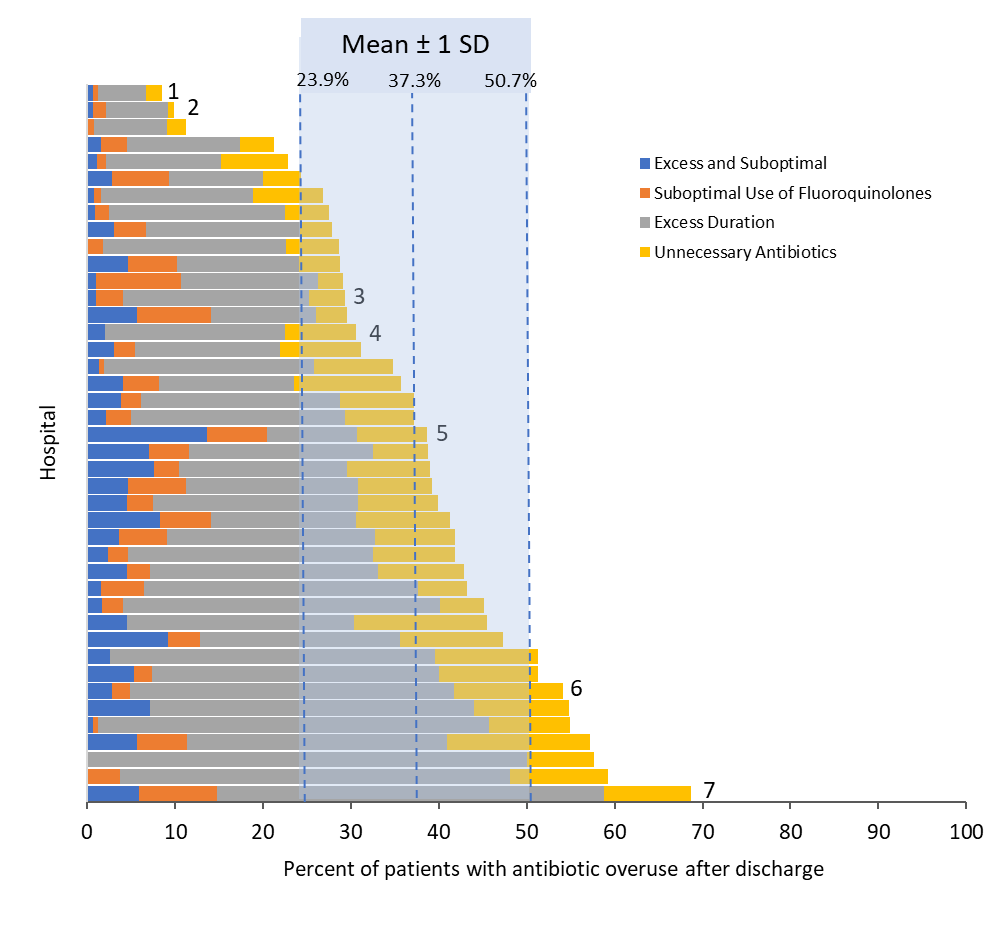


Hospital rankings, 1 (highest performing) to 7 (lowest performing), are presented on graph. Note that hospitals classified as medium performing had antibiotic overuse within ± 1 standard deviation of the mean (blue overlay on figure), and high and low performing hospitals were > 1 standard deviation below and above the mean, respectively.

Overuse Definitions: Unnecessary Antibiotics, if no radiographs in first 48 hours of hospitalization were either consistent or potentially consistent with pneumonia OR if the patient had fewer than 2 clinical signs of symptoms of pneumonia;

Excess Duration, duration was > 1 day longer than expected duration, based on clinical stability;

Suboptimal Use of Fluoroquinolones, use of fluoroquinolone when an acceptable alternative was available;

Excess and Suboptimal, suboptimal use of fluoroquinolones (as defined above) AND excess duration (as defined above)

# **eTable 1**. Interview and Survey Response Rates, by Hospital

|  | High Performing | High Performing | Medium Performing | Medium Performing | Medium Performing | Low Performing | Low Performing |
| --- | --- | --- | --- | --- | --- | --- | --- |
| Interview Participation Rate (participated/invited) | | | | | | | |
| Total | 100% (16/16) | 100% (15/15) | 88% (15/17) | 93% (14/15) | 73% (8/11) | 100% (15/15) | 70% (7/10) |
| Antibiotic Stewardship leaders | 2/2 | 2/2 | 2/2 | 2/2 | 2/2^f^ | 2/2 | 1/2 |
| Hospital leaders | 2/2 | 2/2 | 2/2 | 1/1 | 2/2 | 2/2 | 1/1* |
| Frontline clinicians | 6/6 | 6/6* | 6/6* | 5/6* | 1/3* | 6/6 | 1/3 |
| Hospitalists | 5/5 | 6/6* | 4/4* | 5/6* | 1/3* | 5/5 | 1/3 |
| Advanced Practice Clinicians | 1/1 | 0/0 | 1/1 | 0/0 | 0/0 | 1/1 | 0/0 |
| Medicine residents | 0/0 | 0/0 | 1/1 | 0/0 | 0/0 | 0/0 | 0/0 |
| Clinical pharmacists | 6/6 | 5/5 | 5/6 | 6/6 | 3/4 | 5/5 | 4/4 |
| Survey Participation Rate (participated/invited) | | | | | | | |
| Total | 100% (16/16) | 93% (14/15) | 93% (14/15) | 93% (13/14) | 88% (7/8) | 100% (15/15) | 100% (7/7) |
| Survey Non-responder; group (n) | n/a | Hospitalist (1) | Hospitalist (1) | Hospitalist (1) | Hospitalist (1) | n/a | n/a |

# **eTable 2.** Interview Responder Characteristics, by Hospital

|  | **High**  **Performing**  (n=16) | **High**  **Performing**  (n=14) | **Medium Performing**  (n=14) | **Medium Performing**  (n=13) | **Medium Performing**  (n=7) | **Low**  **Performing**  (n=15) | **Low**  **Performing**  (n=7) |
| --- | --- | --- | --- | --- | --- | --- | --- |
| **All** | | | | | | | |
| Years since finished terminal training^a^,  Median (IQR) | 7.5  (4.5, 10+) | 9.3  (4.5, 10+) | 6.5  (3.0, 9.6) | 10+  (4.5, 10+) | 6.5  (4.5, 10+) | 6.5  (3.5, 10+) | 6.5  (2.5, 8.3) |
| Work at multiple hospitals, n (%) | 3 (19%) | 3 (21%) | 2 (14%) | 1 (8%) | 4 (57%) | 1 (7%) | 0 |
| Sex, n (%) |  |  |  |  |  |  |  |
| Female | 11 (69%) | 8 (57%) | 6 (43%) | 3 (23%) | 3 (43%) | 5 (33%) | 3 (43%) |
| Male | 5 (31%) | 6 (43%) | 8 (57%) | 10 (77%) | 4 (57%) | 10 (67%) | 4 (57%) |
| Race, n (%) |  |  |  |  |  |  |  |
| Asian | 4 (25%) | 0 | 2 (14%) | 2 (15%) | 1 (14%) | 0 | 0 |
| Black/ African American | 0 | 0 | 0 | 0 | 0 | 0 | 0 |
| White/ Caucasian | 11 (69%) | 13 (93%) | 11 (79%) | 11 (85%) | 6 (86%) | 15 (100%) | 6 (86%) |
| Other/ Prefer Not to Disclose | 1 (6%) | 1 (7%) | 1 (7%) | 0 | 0 | 0 | 1 (14%) |
| Ethnicity, n (%) |  |  |  |  |  |  |  |
| Hispanic/ Latino | 2 (13%) | 0 | 0 | 0 | 1 (14%) | 0 | 0 |
| Non-Hispanic/ Latino | 14 (88%) | 14 (100%) | 13 (93%) | 13 (100%) | 5 (71%) | 15 (100%) | 6 (86%) |
| None Selected | 0 | 0 | 1 (7%) | 0 | 1 (14%) | 0 | 1 (14%) |
| **ASP Leaders** | | | | | | | |
| Specialty (n) | ID Physician (1)  ID Pharmacist (1) | ID Physician (1)  ID Pharmacist (1) | ID Physician (1)  ID Pharmacist (2) | ID Physician (1) Non-ID Pharmacist (1) | ID Pharmacist^b^ (2) | ID Physician (1)  Non-ID Pharmacist (1) | Non-ID Pharmacist (1) |
| **Hospital Leaders** | | | | | | | |
| Title (n) | Senior Pharmacy Director (1)  Chief Medical Epidemiologist (1) | Chief Quality Officer (1)  Chief Medical Officer (1) | Senior Medical Director^b^ (1)  Pharmacist Manager of ASP^b^ (1) | Director of Quality (1) | Medical Director, ID Tele-health^b^ (1)  Infection Prevention Director^b^ (1) | Director, Quality and Patient Safety^b^ (1)  Infection Prevention nurse (1) | Pharmacy Director (1) |
| **Hospitalists** | | | | | | | |
| Title (n) | Hospitalist (5)  APP (1) | Hospitalist (6) | Hospitalist (5)  Resident (1)  APP (1) | Hospitalist (5) | Hospitalist (1) | Hospitalist (5)  APP (1) | Hospitalist (1) |
| Private^b^, n (%) | 0 (0) | 0 (0) | 0 (0) | 1 (14%) | 0 (0) | 6 (100%) | 0 (0) |
| **Clinical Pharmacists** | | | | | | | |
| Title (n) | Pharmacist (6) | Pharmacist (5) | Pharmacist (4) | Pharmacist (5)  Pharmacy resident (1) | Pharmacist (3) | Pharmacist (5) | Pharmacist (4) |

^a^ Based on categorical variable: ≤1 year, 1 to 3 years, 4 to 5 years, 6 to 7 years, 8 to 9 years, ≥10 years

^b^ System-level employment

All interviewees were employed by the hospital or a hospital-associated group practice with the exception of hospitalists, some of whom reported private employment. Some hospitals also had ID physicians who were private practice but they were not interviewed.

Abbreviations: ASP, antibiotic stewardship program; ID, infectious diseases; APP, Advanced practice practitioner

# eTable 3. Antibiotic Stewardship Characteristics and Infrastructure, by Hospital Performance

|  | High Performing | High Performing | | Medium Performing | Medium Performing | | Medium Performing | Low Performing | | Low Performing | | |
| --- | --- | --- | --- | --- | --- | --- | --- | --- | --- | --- | --- | --- |
| Tier One Items – Critical Infrastructure (System Level) | | | | | | | | | | | | |
| Stewardship resources increased since Joint Commission standard | Yes | Yes | | Yes | No | | Yes | No | | Yes | | |
| Hospital policy requiring documentation of intended antibiotic duration | Yes | No | | Yes | Yes | | Yes | Yes | | No | | |
| Institutional treatment guideline for UTI** | Developing | Yes | | Yes | Developing | | No | Developing  (Currently use a regional antibiogram developed outside system) | | No | | |
| Indications of obtaining urine culture | N/A | Yes | | Yes | N/A | | N/A | N/A | | N/A | | |
| Recommendations for not treating ASB | N/A | Yes | | Yes | N/A | | N/A | N/A | | N/A | | |
| Antibiotic regimens concordant with national guidelines | N/A | Yes | | Yes | N/A | | N/A | N/A | | N/A | | |
| Recommend against FQ as first line agent for cystitis | N/A | Yes | | Yes | N/A | | N/A | N/A | | N/A | | |
| Education on UTI and ASB | Yes | Yes | | Yes | Yes | | Yes | Yes | | Yes | | |
| Institutional treatment guideline for PNA** | No; but robust orderset with decision-support | Yes | | Yes | Yes | | No | Developing  (Currently use a regional antibiogram developed outside system) | | No | | |
| Antibiotic regimens consistent with national guidelines | Yes | Yes | | Yes | N/A | | Yes | N/A | | Yes | | |
| Recommends 5-day treatment for CAP | Yes | Yes | | No (7) | N/A | | No (7) | N/A | | No (7) | | |
| FQ as first line agent for CAP w/o PCN allergy | No | No | | No | N/A | | No | N/A | | No | | |
| PNA oral step-down recommendations | Yes | Yes | | Yes | N/A | | Yes | N/A | | Yes | | |
| Recommendation for de-escalation | Yes | Yes | | Yes | N/A | | Yes | N/A | | Yes | | |
| Education on Pneumonia | Yes | Yes | | Yes | Yes | | Yes | Yes | | Yes | | |
| Tier Two Items – Inpatient Antibiotic Stewardship (System Level) | | | | | | | | | | | | |
| Antibiotic timeout at 48 to 72 hours | Yes | No | Yes | | | Yes | Yes | | Yes | | | No |
| Fluoroquinolone restriction | No | Yes | Some | | | No | Some | | No | | | No |
| Number of FQ Interventions | 3 | 4 | 4 | | | 3 | 4 | | 3 | | | 0 |
| Tracked rates of FQ | No | Yes | Yes | | | Yes | Yes | | Yes | | | No |
| Timeout with FQ | Yes | No | No | | | No | No | | Yes | | | No |
| Provider feedback on FQ rates | No | Yes | Yes | | | No | Yes | | No | | | No |
| Education | Yes | Yes | Yes | | | Yes | Yes | | Yes | | | No |
| Guidelines | Yes | Yes | Yes | | | Yes | Yes | | No | | | No |
| Diagnostic stewardship interventions | 3 | 1 | 1 | | | 0 | 1 | | 3 | | | 0 |
| Removal/change of urine culture from pre-op ordersets | Yes | Yes | Yes | | | No | Yes | | No | | | No |
| Removal/change of urine culture from ED ordersets | Yes | No | No | | | No | No | | Yes | | | No |
| Removal/change of urine culture – admission ordersets | No | No | No | | | No | No | | No | | | No |
| Removal/change urine culture testing – other ordersets | No | No | No | | | No | No | | No | | | No |
| Added reflex testing (urinalysis cutoff to urine cultures) | No | No | No | | | No | No | | No | | | No |
| Removed reflex test (urinalysis cutoff to urine cultures) | No | No | No | | | No | No | | No | | | No |
| Hiding urine culture results in some settings | No | No | No | | | No | No | | No | | | No |
| Requiring MD order to run urine cultures in ED | No | No | No | | | No | No | | Yes | | | No |
| Other 2-step urine culture initiatives to reduce ED culture | Yes | No | No | | | No | No | | No | | | No |
| Framing urine culture results in test results | No | No | No | | | No | No | | Yes | | | No |
| Audit and feedback for UTI | Yes | Yes | Yes | | | No | Yes | | Yes | | | Yes |
| Audit and feedback for ASB | Yes | Yes | Yes | | | No | Yes | | Yes | | | Yes |
| Computerized provider order entry (CPOE) for UTI | No | No | Yes | | | Yes | Yes | | No | | | No |
| CPOE for ASB | No | No | Yes | | | No | Yes | | No | | | No |
| Preset duration of antibiotics for patients with pneumonia | Yes | No | Yes | | | Yes | Yes | | Yes | | | No |
| Audit and feedback for pneumonia | Yes | Yes | Yes | | | Yes | Yes | | Yes | | | Yes |
| CPOE for pneumonia | Yes | Yes | Yes | | | Yes | Yes | | Yes | | | Yes |
| Tier Three Items – Discharge Specific Stewardship Interventions | | | | | | | | | | | | |
| Discharge intervention de-emphasizing FQ | Yes | Yes | Yes | | | No | No | | No | | No | |
| Antibiotic use data on discharge antibiotics | Yes | No | No | | | No | No | | No | | No | |
| Review of outpatient antibiotics before discharge | No | Yes | No | | | No | No | | No | | No | |
| Total Number of ROAD Home Interventions | | | | | | | | | | | | |
| Tier 1 | 4 | 5 | 6 | | | 4 | 4 | | 3 | | 3 | |
| Tier 2, unweighted (weighted) | 12 (24) | 10 (20) | 13 (26) | | | 8 (16) | 13 (26) | | 12 (24) | | 4 (8) | |
| Tier 3, unweighted (weighted) | 2 (6) | 2 (6) | 1 (3) | | | 0 | 0 | | 0 | | 0 | |
| Sum | 18 | 17 | 20 | | | 12 | 17 | | 15 | | 7 | |

* On 01/01/17, the Joint Commission launched a new standard for hospitals, critical access hospitals, and nursing care centers that addresses anti-microbial stewardship. Since 01/01/17, have stewardships resources increased at your hospital?

**Among respondents indicating that they have an institutional treatment guideline and answered “Yes and it is a new guideline or has been updated in the past 12 months”

# **eTable 4.** Summary of Vignette Responses, by Hospital Performance

|  | High Performing | High Performing | Medium Performing | Medium Performing | Medium Performing | Low Performing | Low Performing |
| --- | --- | --- | --- | --- | --- | --- | --- |
| **Vignette 1 – Typical CAP**: Patient is a healthy, 65-year-old woman admitted with community-acquired pneumonia (right upper lobe infiltrate, cough, fever) who is improving well and ready for discharge. She’s received three days of ceftriaxone in the hospital.  **Correct Answer:** We were looking for a 5-day total antibiotic duration (though accepted 3-day duration) and avoidance of fluoroquinolones. | | | | | | | |
| Chose appropriately short ≤5-day antibiotic duration, n (%) | | | | | | | |
| All Respondents | 14/14 (100%) | 12/12 (100%) | 10/12 (83%) | 11/12 (92%) | 3/5 (60%) | 12/13 (92%) | 4/6 (67%) |
| Pharmacists | 6/6 (100%) | 5/5 (100%) | 4/4 (100%) | 5/6 (83%) | 1/3 (33%) | 4/5 (80%) | 2/4 (50%) |
| Hospitalists | 6/6 (100%) | 5/5 (100%) | 3/5 (60%) | 4/4 (100%) | n/a | 6/6 (100%) | 1/1 (100%) |
| ASP Leaders | 2/2 (100%) | 2/2 (100%) | 3/3 (100%) | 2/2 (100%) | 2/2 (100%) | 2/2 (100%) | 1/1 (100%) |
| Selected correct antibiotic (avoiding fluoroquinolone), n (%) | | | | | | | |
| All Respondents | 14/14 (100%) | 12/12 (100%) | 12/12 (100%) | 12/12 (100%) | 5/5 (100%) | 13/13 (100%) | 4/6 (67%) |
| Pharmacists | 6/6 (100%) | 5/5 (100%) | 4/4 (100%) | 6/6 (100%) | 3/3 (100%) | 5/5 (100%) | 2/4 (50%) |
| Hospitalists | 6/6 (100%) | 5/5 (100%) | 5/5 (100%) | 4/4 (100%) | n/a | 6/6 (100%) | 1/1 (100%) |
| ASP Leaders | 2/2 (100%) | 2/2 (100%) | 3/3 (100%) | 2/2 (100%) | 2/2 (100%) | 2/2 (100%) | 1/1 (100%) |
| Additional Comments | | | | | | | |
|  | - 100% cefuroxime - Some recommended 3-day duration based on new clinical trial | - Though all correct, selection varied. | - Hospitalists and ASP varied on selection and duration. - System guidelines allow 5 to 7-day duration. | - Pharmacists varied on selection - Hospitalists and ASP consistent. | - Pharmacists and ASP varied on duration and selection - System guidelines allow 5 to 7-day duration | - Hospitalists, pharmacists, and ASP varied on selection | - 2 of 4 of pharmacists chose a FQ |
| **Vignette 2 – CAP treated as HCAP**: Patient is a healthy, 65-year-old woman admitted with pneumonia (right upper lobe infiltrate, cough, fever) who had a MRSA pneumonia six months previously. She was recently hospitalized with a STEMI with a successful PCI. This hospitalization, she was unable to produce sputum for analysis. Her MRSA nares is negative, and she is improving well and is ready for discharge. She's received three days of vancomycin and Zosyn in the hospital.  **Correct Answer:** We were looking for a 5-day total antibiotic duration (though accepted 3-day duration) and avoidance of fluoroquinolones. | | | | | | | |
| Chose appropriately short ≤5-day antibiotic duration, n (%) | | | | | | | |
| All Respondents | 12/14 (86%) | 9/12 (75%) | 7/12 (58%) | 8/12 (67%) | 3/5 (60%) | 8/13 (62%) | 1/6 (17%) |
| Pharmacists | 6/6 (100%) | 4/5 (80%) | 1/4 (25%) | 5/6 (83%) | 1/3 (33%) | 3/5 (60%) | 0/4 (0%) |
| Hospitalists | 4/6 (67%) | 3/5 (60%) | 3/5 (60%) | 1/4 (25%) | n/a | 3/6 (50%) | 0/1 (0%) |
| ASP Leaders | 2/2 (100%) | 2/2 (100%) | 3/3 (100%) | 2/2 (100%) | 2/2 (100%) | 2/2 (100%) | 1/1 (100%) |
| Selected correct antibiotic (avoiding fluoroquinolone), n (%) | | | | | | | |
| All Respondents | 12/14 (86%) | 9/12 (75%) | 12/12 (100%) | 10/12 (83%) | 3/5 (60%) | 10/13 (77%) | 3/6 (50%) |
| Pharmacists | 5/6 (83%) | 5/5 (100%) | 4/4 (100%) | 5/6 (83%) | 1/3 (33%) | 3/5 (60%) | 1/4 (25%) |
| Hospitalists | 5/6 (83%) | 2/5 (40%) | 5/5 (100%) | 3/4 (75%) | n/a | 5/6 (83%) | 1/1 (100%) |
| ASP Leaders | 2/2 (100%) | 2/2 (100%) | 3/3 (100%) | 2/2 (100%) | 2/2 (100%) | 2/2 (100%) | 1/1 (100%) |
| Additional Comments | | | | | | | |
|  | - 3-7 days - Mostly cefuroxime, two suggested FQ | - Pharmacists and hospitalists varied on selection and duration - Hospitalists more inconsistent than pharmacists - 2 selected FQ; 1 linezolid | - Pharmacists, hospitalists, and ASP varied on selection and duration - System guidelines allow 5 to 7-day duration | - Pharmacists and hospitalists varied on selection and duration - 2 selected FQ - Still use HCAP term | - Pharmacists and ASP varied on duration and selection - 2 selected FQ | - Hospitalists, pharmacists, and ASP varied on selection and duration - 3 selected FQ and/or linezolid | - Pharmacists varied on duration and selection - 2 selected FQ; 1 vancomycin - Still use HCAP term |
| **Vignette 3 – UTI with Sepsis**: The patient is a healthy, 65-year-old woman admitted with hypotension, fever, dysuria, and a urine culture growing pan-sensitive *E. coli*. The patient is improving and ready for discharge. She's received three days of ceftriaxone in the hospital.  **Correct Answer:** The fever and hypotension should make you suspect that this patient has pyelonephritis or UTI with systemic signs of infection. Typically, when these patients respond quickly, the recommended duration is 7 days. | | | | | | | |
| Correct antibiotic duration, n (%) | | | | | | | |
| All Respondents | 3/14 (21%) | 4/12 (33%) | 8/12 (67%) | 7/12 (58%) | 1/5 (20%) | 6/13 (46%) | 3/6 (50%) |
| Pharmacists | 0/6 (0%) | 2/5 (40%) | 3/4 (75%) | 2/6 (33%) | 0/3 (0%) | 3/5 (60%) | 1/4 (25%) |
| Hospitalists | 3/6 (50%) | 1/5 (20%) | 3/5 (60%) | 4/4 (100%) | n/a | 1/6 (17%) | 1/1 (100%) |
| ASP Leaders | 0/2 (0%) | 1/2(50%) | 2/3 (67%) | 1/2 (50%) | 1/2 (50%) | 2/2 (100%) | 1/1 (100%) |
| Correct antibiotic selection, n (%) | | | | | | | |
| All Respondents | 4/4 (100%) | 4/6 (67%) | 8/11 (73%) | 7/10 (70%) | 3/3 (100%) | 10/11 (91%) | 3/4 (75%) |
| Pharmacists | n/a | 3/3 (100%) | 2/3 (67%) | 3/5 (60%) | 1/1 (100%) | 4/4 (100%) | 1/2 (50%) |
| Hospitalists | 4/4 (100%) | 1/2 (50%) | 4/5 (80%) | 3/4 (75%) | n/a | 5/5 (100%) | 1/1 (100%) |
| ASP Leaders | n/a | 0/1 (0%) | 2/3 (67%) | 1/1 (100%) | 2/2 (100%) | 1/2 (50%) | 1/1 (100%) |
| Additional Comments | | | | | | | |
|  | - 11 of 14 respondents recommended undertreatment - Hospitalists varied on selection | - 8 of 12 respondents recommended undertreatment - All groups similarly inconsistent - 1 selected a FQ | - 4 of 12 respondents recommended undertreatment - Pharmacists, hospitalists, and ASP varied on selection - 1 selected a FQ | - 1 of 2 ASP and 4 of 6 pharmacists recommended undertreatment - All groups varied on selection | - 4 of 5 of respondents recommended undertreatment - Pharmacists and ASP varied on selection | - 5 of 6 hospitalists and 2 of 5 pharmacists recommended undertreatment - Hospitalists and ASP varied on selection | - 3 of 4 pharmacists recommended undertreatment |
| **Vignette 4 – Stable Altered Mental Status**: The patient is a healthy, 65-year-old woman with dementia (and no other conditions) admitted with altered mental status. Vital signs remained within normal limits. Her urine culture grew pan-sensitive *E. coli*. All other work-up has been negative. She was treated with ceftriaxone for three days, IV fluids, and her Ultram was held. She has improved and is ready for discharge. She denies urinary symptoms.  **Correct Answer:** This patient had hemodynamically stable altered mental status and asymptomatic bacteriuria. IDSA guidelines^4^ recommend watchful waiting before starting antibiotics. | | | | | | | |
| Stop antibiotics at discharge, n (%) | | | | | | | |
| All Respondents | 13/14 (93%) | 11/12 (92%) | 10/12 (83%) | 9/12 (75%) | 5/5 (100%) | 12/13 (92%) | 6/6 (100%) |
| Pharmacists | 6/6 (100%) | 4/5 (80%) | 4/4 (100%) | 4/6 (67%) | 3/3 (100%) | 5/5 (100%) | 4/4 (100%) |
| Hospitalists | 5/6 (83%) | 5/5 (100%) | 3/5 (60%) | 3/4 (75%) | n/a | 5/6 (83%) | 1/1 (100%) |
| ASP Leaders | 2/2 (100%) | 2/2 (100%) | 3/3 (100%) | 2/2 (100%) | 2/2 (100%) | 2/2 (100%) | 1/1 (100%) |
| **Vignette 5 – Unstable Altered Mental Status**: The patient is a healthy, 65-year-old woman with dementia (and no other conditions) admitted with altered mental status. On admission, her heart rate was 120, blood pressure 80/40, respiratory rate 24, temperature 38.5 C. Her urine culture grew pan-sensitive *E. coli*. All other work-up has been negative. She was treated with ceftriaxone for three days, given IV fluids, and her Ultram was held. She has now improved; her vital signs have normalized, and she denies urinary symptoms.  **Correct Answer:** This patient had hemodynamically **un**stable altered mental status and bacteriuria. IDSA guidelines recommend empiric treatment with antibiotics while looking for other causes. There is equipoise as to whether this patient did vs. did not have a UTI, thus we would have accepted no antibiotic treatment (i.e., “treating as ASB”) or a maximum 7-day antibiotic course (4 days at discharge), avoiding FQs. | | | | | | | |
| Treated as ASB, n (%) | | | | | | | |
| All Respondents | 9/14 (64%) | 2/12 (17%) | 5/12 (42%) | 4/12 (33%) | 3/5 (60%) | 6/13 (46%) | 3/6 (50%) |
| Pharmacists | 5/6 (83%) | 0/5 (0%) | 4/4 (100%) | 2/6 (33% | 3/3 (100%) | 3/5 (60%) | 3/4 (75%) |
| Hospitalists | 2/6 (33%) | 2/5 (40%) | 0/5 (0%) | 1/4 (25%) | n/a | 2/6 (33%) | 0/1 (0%) |
| ASP Leaders | 2/2 (100%) | 0/2 (0%) | 1/3 (33%) | 1/2 (50%) | 0/2 (0%) | 1/2 (50%) | 0/1 (0%) |
| Correct antibiotic selection (if treated as UTI), n (%) | | | | | | | |
| All Respondents | 5/5 (100%) | 8/10 (80%) | 6/7 (86%) | 8/8 (100%) | 2/2 (100%) | 8/8 (100%) | 2/2 (100%) |
| Pharmacists | 1/1 (100%) | 5/5 (100%) | n/a | 4/4 (100% | n/a | 3/3 (100%) | n/a |
| Hospitalists | 4/4 (100%) | 2/3 (67%) | 4/5 (80%) | 3/3 100%) | n/a | 4/4 (100%) | 1/1 (100%) |
| ASP Leaders | n/a | 1/2 (50%) | 2/2 (100%) | 1/1 (100%) | 2/2 (100%) | 1/1 (100%) | 1/1 (100%) |
| Additional Comments | | | | | | | |
|  | - Hospitalists varied on selection and duration | - Within hospital respondent group variation on duration and selection - 2 selected a FQ | - Within hospital respondent group variations on duration - 1 selected a FQ | - Within hospital respondent group variations on duration and selection | - Varied on duration | - Within hospital respondent group variations on duration | - Within hospital respondent group variations on duration |
| Summary | | | | | | | |
| Total Correct Responses | 86/102 (84.0%) | 71/90 (82.1%) | 78/95 (82.1%) | 76/94 (80.9%) | 28/38 (73.7%) | 85/104 (81.7%) | 29/45 (64.4%) |
| Discharge Duration, mean (SD) | 1.2 (1.5) | 1.8 (1.5) | 2.2 (1.7) | 2.2 (1.9) | 1.8 (2.6) | 1.9 (1.5) | 2.8 (3.1) |
| Fluoroquinolone Use (%) | 2/70 (2.9%) | 5/60 (8.3%) | 3/60 (5.0%) | 3/60 (5.0%) | 2/25 (8.0%) | 2/65 (3.1%) | 5/30 (15.7%) |
| ASP Leaders with Consistent Answers | 4/5 (80%) | 3/5 (60%) | 1/5 (20%) | 3/5 (60%) | 1/5 (20%) | 1/5 (20%) | N/A (only 1 ASP) |

Abbreviations: CAP, community-acquired pneumonia; ASP, antibiotic stewardship program; FQ, fluoroquinolone; HCAP, healthcare-associated pneumonia; MRSA, Methicillin-resistant Staphylococcus aureus; STEMI, ST-elevation myocardial infarction; PCI, percutaneous coronary intervention; UTI, urinary tract infection; ASB, asymptomatic bacteriuria; *E. coli*., *Escherichia coli*; IDSA, Infectious Diseases Society of America; SD, standard deviation

| eTable 5. Pharmacist Survey Responses, by Hospital Performance | | | | | | | |  |
| --- | --- | --- | --- | --- | --- | --- | --- | --- |
|  | High Performing (n=6) | High Performing (n=5) | Medium Performing (n=5) | Medium Performing (n=6) | Medium  Performing (n=3) | Low Performing (n=5) | Low Performing (n=4) | Correlation^1^  r (p-value) |
| How important do you think antibiotic stewardship is? (1=very unimportant, 5=very important) | | | | | | | | |
|  | 4.7 | 4.0 | 5.0 | 4.0 | 4.7 | 5.0 | 4.7 | 0.23 (0.199) |
| For the following questions, please rate how strongly you agree or disagree, where: 1=strongly disagree; 2=disagree; 3=neither agree/disagree; 4=agree; 5=strongly agree; mean values shown | | | | | | | | |
| I am respected by my pharmacist colleagues. | 4.5 | 4.8 | 4.6 | 4.5 | 4.7 | 4.4 | 4.0 | -0.24 (0.173) |
| I am respected by my hospitalist colleagues. | 4.7 | 5.0 | 4.6 | 4.2 | 4.7 | 3.6 | 4.0 | -0.55 (0.001) |
| I am considered a valuable member of the clinical team. | 4.7 | 5.0 | 4.6 | 4.5 | 4.3 | 4.0 | 4.0 | -0.54 (0.001) |
| How comfortable are you recommending changes to antibiotic prescriptions if you think guidelines are not being met? 1=very uncomfortable; 5=very comfortable | 4.7 | 4.6 | 5.0 | 4.3 | 4.0 | 4.4 | 4.0 | -0.41 (0.020) |

^1^ The association between pharmacist survey responses and hospital performance rank was assessed using Spearman correlation with p-value <0.05 considered statistically significant; negative r value indicates lower numbers as performance worsens

# eTable 6. Joint Display Describing 4 Major Themes from the Mixed-Methods Data Integration

| Themes | Survey Findings | Findings from Data and Object Review | Qualitative Findings | Synthesis of Findings |
| --- | --- | --- | --- | --- |
| Knowledge/Comfort with Antibiotic Stewardship | - Pharmacist comfort recommending antibiotic changes lower with worse performance (Spearman rho -0.41, p<0.05)   Vignettes:   - Antibiotic duration and fluoroquinolone use increased as performance decreased - High performing hospitals were more likely to err on the side of undertreatment - ASP leaders’ responses were more consistent in high performing hospitals | - Institution-specific guidelines were less likely to exist at lower performers; when they did exist, medium- and low-performing hospitals had out of date guidelines (e.g., still used the term HCAP) or guidelines using ranges for duration (e.g., 5-7 days for CAP) | - Pharmacists at high-performing institutions were more likely to speak of antibiotic stewardship as a core element of their job - Pharmacists at high-performing institutions reported using primary literature to support their position if disagreements arose - Pharmacists at low-performing institutions had less post-graduate training including stewardship-specific training | Antibiotic knowledge, comfort, and ownership of stewardship was less consistent at lower-performing hospitals, particularly among pharmacists, which could be driven by lack of institution-specific guidelines and tools, lack of training, or lack of access to ID expertise |
| Inter-professional Dynamics, Group Cohesiveness | Pharmacist responses to the following questions were correlated with performance:   - I am respected by my hospitalist colleagues (Spearman rho -0.55, p<0.001) - I am considered a valuable member of the clinical team (Spearman rho -0.54, p<0.001) | - High-performing hospitals were more likely to have pharmacists participate in face-to-face rounds - Private physicians were more common in medium and low-performing hospitals - One high-performing hospital incentivized meeting attendance in hospitalist quality bonuses. At least one low performing hospital reported RVU based incentives. | - Though generally relationships were described as positive across performance groups, pharmacists at low-performing hospitals more often discussed difficulties dealing with clinicians - Absolute respect for “physician autonomy” was more commonly brought up as a barrier in low performing hospitals - At low performing hospitals, pharmacists often communicated with hospitalists through an intermediary - At low performing hospitals, hospitalists would often skip inter-disciplinary rounds or were otherwise unengaged in team-based care - Pharmacists at high-performing hospitals noted less “pushback” from clinicians and were more likely to report team-based and evidence-based approaches to decision-making; in contrast, pharmacists at low-performing hospitals reported “picking their battles” | Relationships were generally positive across all performance categories; however, respect for pharmacists appeared higher at high-performing hospitals. Similarly, interdisciplinary teams with engaged hospitalists were more common at high performing hospitals.  High-performing hospitals were more likely to have pharmacists participate in face-to-face rounds which was viewed by all as a way to have bi-directional conversations and forge relationships. |
| Tools and Infrastructure | N/A | - High performing hospitals had more infrastructure and resources overall and were more likely to be stewardship flagship for system - Number of stewardship interventions was correlated with performance - Pneumonia ordersets were more robust at high-performing hospitals - High performing hospitals were more likely to report care transition infrastructure (e.g., meds to bed, transition of care pharmacists) | - High performing hospitals had robust guidelines that were integrated across decision-support tools and were heavily used by pharmacists - Hospitalists did not use institutional guidelines regardless of performance, but at high performing hospitals the guidelines were operationalized via ordersets - The highest performing hospital designed their orderset with buy-in from frontline clinicians, making it commonly used - Data feedback was a common tool at high performing institutions; for example, the highest performing institutions sent individual feedback letters to clinicians when they over-prescribed antibiotics | The more stewardship interventions and the more integrated in other tools, the better antibiotic use will be.  Hospitalist do not refer to guidelines but engaging them during orderset development can promote orderset use and help operationalize guidelines. Pharmacists rely on guidelines to support their recommendations. |
| ID Physician Role | - ID physician at lowest performing hospital did not answer survey | - High performing hospitals had more FTE designated for ID physicians to participate in stewardship and were more likely to have on-site ID physicians | - Engaged and strong ID physician leadership was present in all high and medium performers - One high performing hospital required all ID physicians to be on the stewardship team - One medium-performing hospitals used required ID consults to promote stewardship goals - The lowest performing hospital’s ID physician’s recommendations were often contrary to stewardship goals - Pharmacists often mentioned that having an ID physician make a recommendation carried more weight - At one medium-performing hospital, it was noted that ID did not “own” pneumonia guidelines and therefore could not incorporate stewardship guidance - At one medium-performing hospital, there was a private ID group that others did not enjoy engaging with, impacting treatment and stewardship when that group was covering | ID physicians are critical for championing stewardship and can be major barriers if they do not engage with or disagree with stewardship principals |

Abbreviations: ASP, antibiotic stewardship program; HCAP, healthcare associated pneumonia; CAP, community-acquired pneumonia; ID, infectious diseases; RVU, relative value unit (i.e., productivity-based incentive); FTE, full-time equivalents
